# Protic Ionic Liquid–H_2_O Mixtures—Structure, Interactions, and Structure–Property Relationships

**DOI:** 10.1021/acs.jpcb.5c07863

**Published:** 2026-01-27

**Authors:** Liisa-Maria Kaljusmaa, Katarzyna Maria Dziubinska-Kuehn, Balázs Erdös, Diandra Tubli, Sander Lillepea, Indrek Reile, Oliver Järvik

**Affiliations:** † Department of Energy Technology, 54561 Tallinn University of Technology, Ehitajate tee 5, Tallinn 19086, Estonia; ‡ 113902 National Institute of Chemical Physics and Biophysics, Akadeemia tee 23, Tallinn 12618, Estonia; § Department of Data Science and Knowledge Discovery, 571514 Simula Metropolitan Center for Digital Engineering, Stensberggata 27, Oslo 0170, Norway

## Abstract

A better understanding of the molecular interactions and, consequently, the structure–property relationships in protic ionic liquids (PIL) can help create more accurate property prediction models and enhance the efficiency of their various applications. This approach is especially relevant in understanding the PIL–H_2_O mixtures. Depending on the application, water molecules can be considered as not only an impurity in PILs but also a dopant or even a cosolvent. Our study investigated density, viscosity, electrical conductivity, and derived properties like thermal expansion coefficients, excess molar volume, and excess viscosity of low-toxic alkanolammonium- and carboxylate-based PIL–H_2_O mixtures. One- (1D) and two-dimensional (2D) nuclear magnetic resonance spectroscopy was employed to elucidate the distribution of water molecules within the PIL structure. In addition, critical aggregation concentrations (CAC) of PIL–H_2_O mixtures were determined based on the physicochemical properties and ^1^H longitudinal relaxation times. The results showed that the strength of PIL–H_2_O interactions depends on the anion, while the cation affects the position of water in the PIL solvent network. Overall, our study provides valuable insight into the molecular modeling and property prediction of this type of PIL, a promising and currently underexplored subclass of ionic liquids.

## Graphical abstract

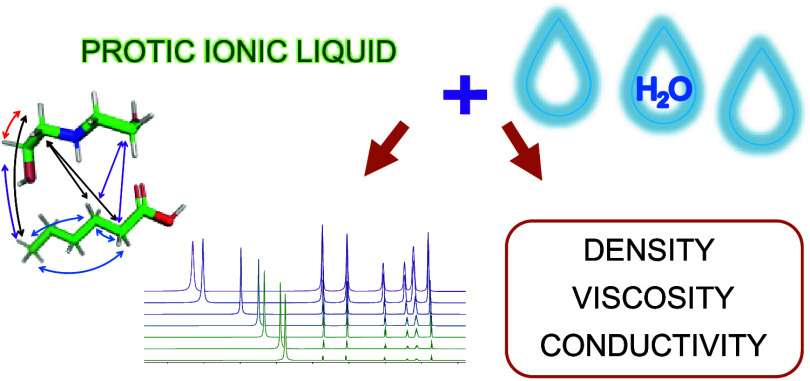

## Introduction

Rapid developments in the fields of computer science and artificial intelligence are continually improving the conditions for simulation and property prediction models, thereby reducing the need for experimental testing. In chemistry, one such area is the investigation of ionic liquids (ILs), in which computational studies and/or the use of machine learning algorithms have been proven to be beneficial for creating a better understanding of and estimating physicochemical properties of these compounds. The ability to predict the physicochemical properties of ILs could potentially provide alternative materials for a broad range of applications, like catalysts, surfactants, or solvents for microextraction.^1–3^ Furthermore, the theoretical approach allows to obtain detailed insights into the noncovalent interactions governing the formation of local microheterogeneities in ILs.^4,5^

In general, ILs are defined as solvents consisting entirely of ions and have a melting point below 100 °C.^6^ They are organic or semiorganic salts comprised of organic cations and organic or inorganic anions.^7^ Consequently, the properties of ILs can be controlled by selecting different combinations of ions, resulting in an estimated number of 10^14^–10^15^ possible ILs according to different sources.^8,9^ Thus, the theoretical possibility to predict their properties, e.g., with the aid of a quantitative structure–property relationship (QSPR) machine learning model(s), could save a lot of resources.^10–12^ However, so far, only around 2500 ILs have been synthesized and to a greater or lesser extent experimentally characterized, with some subclasses such as imidazolium and triflate ILs being investigated more than others.^13^ In theoretical chemistry, this results in some models performing better when working with the types of ILs that were used to establish the parameters of a particular model.^13,14^

Protic ionic liquids (PILs) are a subclass of ILs that are formed by a neutralization reaction between specific Brønsted acids and Brønsted bases. Compared with other molecular liquids, PILs have several unique properties such as nonflammability, low vapor pressure, and high electrical conductivity, which are useful in different application sectors, such as electrolytes, solvents, lubricants, or phase-change materials.^15–18^ Furthermore, it has been demonstrated that QSPR machine learning models can successfully predict the properties of PILs, including viscosity and conductivity, provided that the quantity and quality of the input data are sufficient. Thus, existing computational studies still emphasize the need for additional experimental data, including more information about the local effects of impurities, such as water, to improve the accuracy of predictions.^11^ Therefore, determining the structure–property relationships of ILs and PILs, both experimentally and theoretically, became an ongoing investigation for many researchers in recent years.^19^

Multiple studies on thermophysical properties of low-toxic and easily preparable^20^ ammonium- and carboxylate-based PILs have shown that their structure–property relationships are influenced by intermolecular interactions, sizes of anion and cation, and packing efficiency of the solvent network.^21–29^ For example, the density (*ρ*) decreases if the aliphatic carbon chain becomes longer in the cation, anion, or both.^21–25^ Alternatively, ρ can also increase with the addition of hydroxyl (OH) groups to cation or anion molecules.^26,27^ Viscosity (*η*) can either increase or decrease as the alkyl chain of the anion lengthens depending on the cation. In PILs based on *N*-butyl-2-hydroxy-ethylammonium, higher *η* was measured in the presence of anions with longer alkyl chains.^22^ A similar effect was observed in alkylammonium-,^28^ diethylammonium-, or dibutylammonium-based PILs as well.^25^ Finally, a study comparing ethanolammonium acetate and ethanolammonium hexanoate has shown the *η* measured at 25 °C to be smaller for the latter PIL,^23^ and also for PILs containing 2-hydroxy-*N*-methyl-ethanammonium cation.^29^

The presence of a secondary solvent in PILs can significantly alter their global properties.^30^ Depending on the concentration and also the polarity of the cosolvent, the ion mobility can potentially be affected by changes in the local equilibrium between ionic species.^31^ Among the different dopants paired with PILs, water remains the most versatile and thus is the explored one. Because most of the ILs are known to be hygroscopic to different degrees,^32^ most PILs are rather hydrophilic due to their protic nature. This makes purification and controlling the moisture content of such liquids challenging.^33–35^

It is confirmed that the presence of small amounts of water in ILs or PILs leads to either an isolated distribution of H_2_O within the polar solvent network or the formation of small H_2_O clusters. Previously, such an effect was observed at <0.1 wt % in *N*-butyl-pyrrolidinium bis­(trifluoromethanesulfonyl)­imide PIL,^36^ or at a mole fraction of water *X*_H_2_O_ < 0.5 in 1-ethyl-3-methylimidazolium ethylsulfate IL.^37^ When the water content increases, e.g., between 0.5 < *X*_H_2_O_ < 0.8 in the case of 1-ethyl-3-methylimidazolium ethylsulfate IL, chain-like water aggregates can form.^37^ Finally, when the water mole fraction is larger than *X*_H_2_O_ = 0.95, the PIL ions become freely solvated by the water network, which becomes the primary solvent in the binary mixture.^37^

The position of water in the IL structure depends strongly on the noncovalent interactions between water and IL.^38^ It has been confirmed by investigating water rotational dynamics that water shows a higher degree of self-clustering in hydrophobic ILs. Hydrophilic ILs exhibit a much lower effect on water aggregation due to better mixing of the dopant with its surrounding ions.^39^ Carboxylate PILs are considered particularly hydrophilic due to a highly polar anion that can have strong hydrogen bonds with water.^40,41^ Because of their hydrophilic character, their mixtures with water could potentially become relevant for many applications, such as phase-change materials^42^ in thermal energy storage, electrolytes^38^ in electrochemical energy storage, or as lubricants^43^ in mechanical systems. Thus, it remains crucial to systematically assess the possible influence of different quantities of H_2_O molecules on the structure of the solvent network in PILs, especially in combination with measurement of their physicochemical properties.

The effect of water on *ρ* and *η* was already observed in exemplary alkanolammonium and carboxylate PILs.^23,29^ Overall, the values of both properties decrease as the water content increases.^23,29^ Electrical conductivity (*κ*) is considered a crucial transport property, giving insight into the behavior of water in PILs.^44^ Changes in *κ* can reflect different IL–water interaction regions, including the rising mobility of ions, degradation of IL–water aggregates, critical aggregation concentration (CAC), and infinite solvation of IL cations and anions by neutral water molecules.^44^ Moreover, in alkanolammonium and carboxylate PILs, *κ* decreases with an increase in hydrophobicity of anions.^23,45^ Here, the more hydrophobic anions are considered to be those with longer alkyl chain lengths; e.g., the ionic conductivity (defined as the electrical conductivity due to the motion of ionic charge) is lower in hexanoate PIL than in acetate PIL, both sharing the same alkanolammonium cation. The addition of water usually decreases the *η* of a PIL, and consequently, leads to increased conductivity due to enhanced molecular mobility. However, the extent of the increase in conductivity with additional water depends on the PIL–water interactions since PILs with more hydrophobic anions tend to have lower self-aggregation concentrations.^45^

One- (1D) and two-dimensional (2D) nuclear magnetic resonance (NMR) spectroscopy has been shown to be among the most suitable techniques to study the influence of water on ILs.^44,46,47^ The high sensitivity of ^1^H NMR chemical shifts (CS) to changes in the local environment of protons in both IL cation and anion and also water molecules serves as a direct indicator of hydrogen-bond formation.^48^ At higher water contents, changes in chemical shifts or longitudinal relaxation time (*T*_1_) trends can be additionally used to estimate the critical micelle concentration (CMC) or CAC, at which IL cation and anion begin to associate and form non- or systematic aggregates at low IL concentration, or a regular solvent network at low values of *X*_H_2_O_.^44,49^

In general, alkanolammonium- and carboxylate-based PILs, especially in the presence of water, are not often investigated due to their difficult properties, such as high viscosity,^50^ low electrical conductivity,^50^ melting point possibly above room temperature,^50^ and worse thermal stability according to dynamic thermogravimetric analysis^51^ compared to other more thoroughly characterized ILs. The measurements remain challenging, even if the addition of H_2_O molecules is confirmed to significantly improve the properties of IL.^29^ Herein, we aim to investigate the influence of water on the properties of selected alkanolammonium- and carboxylate-based protic ionic liquids. Four PILs, ethanolammonium acetate (MEAA), ethanolammonium hexanoate (MEAH), diethanolammonium acetate (DEAA), and diethanolammonium hexanoate (DEAH), are selected to represent PILs with variable cation and anion sizes. The densities, viscosities, and electrical conductivities of each PIL at varying temperatures are studied in the presence of water. 1D and 2D NMR analyses are performed to assess the influence of water on the structural arrangement of PILs, verify the hydrogen-bond formation, and determine the CAC for each PIL. The present study establishes the first connection between the trends in the experimentally determined physicochemical and transport properties of PILs and the distribution of water molecules within the solvent network, thereby opening a discussion about their structure–property relationships. It could provide valuable insights into the molecular modeling of PILs, a not-yet-widely-explored but promising subclass of ionic liquids.

## Experimental Section

### Materials

Four protic ionic liquids (PILs) were synthesized using the following chemicals without further purification: monoethanolamine (purity >99 wt %, Fisher Scientific, CAS no. 141-43-5), diethanolamine (99 wt %, ThermoFisher, CAS no. 111-42-2), acetic acid (99 wt %, Fisher Scientific, CAS no. 64-19-7), hexanoic acid (99 wt %, ThermoFisher, CAS no. 142-62-1), methanol (>99 wt %, Honeywell), and deuterium oxide (isotopic purity >99.99%, Sigma-Aldrich). Milli-Q water with resistivity <18.2 M Ω cm at 25 °C was purified using the Milli-Q Integral 10 water purification system, Millipore Corporation.

### Synthesis of Protic Ionic Liquids

Structures of all of the chemical compounds used to synthesize PILs are presented in Figure 1. The synthesized liquids investigated in this work and their initial water contents after purification are listed in Table 1. The PILs were synthesized by an acid–base neutralization reaction.^52^ Monoethanolamine (MEA) and diethanolamine (DEA) were mixed with an equimolar amount of acetic or hexanoic acid to formulate the following PILs: monoethanolammonium acetate (MEAA), diethanolammonium acetate (DEAA), monoethanolammonium hexanoate (MEAH), and diethanolammonium hexanoate (DEAH). In each reaction, the acid was added dropwise to the base with constant stirring in a three-necked round-bottom flask over ice. Synthesis was carried out under continuous flow of the inert gas (N_2_, >99.999% purity). Stirring was continued for at least 23 h at 298 K (room temperature) after the acid had been fully added to the synthesis mixture.

**Figure 1:**
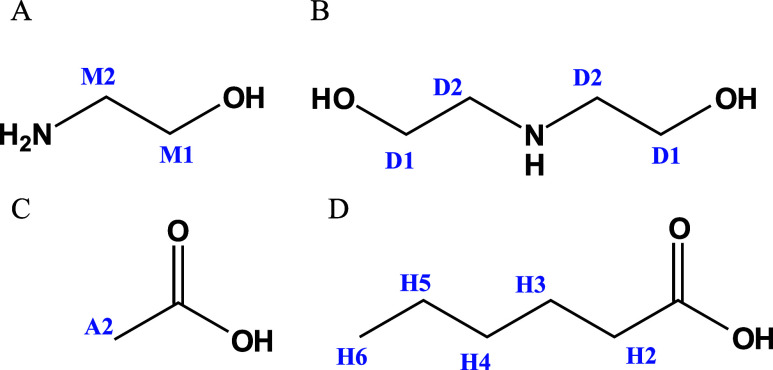
Structures and labels in chemicals used to synthesize the protic ionic liquids: (A) monoethanolamine, MEA (M); (B) diethanolamine, DEA (D); (C) acetic acid (A); and (D) hexanoic acid (H).

**Table 1:** Water Content of Protic Ionic Liquids (PILs) Synthesized in the Study^a^

PIL	abbreviation	water content, ppm
ethanolammonium acetate	MEAA	222 ± 11.1
ethanolammonium hexanoate	MEAH	446 ± 22.3
diethanolammonium acetate	DEAA	1111 ± 55.5
diethanolammonium hexanoate	DEAH	601 ± 30.0

a The water content was assessed using Karl Fischer titration and is presented with a systematic uncertainty of 5%.

Methanol was used as the solvent environment during the reaction to reduce viscosity and enhance mixing. Removal of solvent and traces of unreacted reagents was carried out on a rotary evaporator at 40 °C and under reduced pressure (5.0 mbar) for a maximum of 24 h. Synthesized PILs were further purified by vacuuming the product at 10^–1^ to 10^–2^ mbar for at least 72 h. The final water content in the purified PILs was determined by coulometric Karl Fischer titration (Titroline 7500 KF trace, SI Analytics, Germany) in 20 wt % solutions in methanol with known water content. Results were calculated based on the mass of the mixture and are presented in Table 1. MEAA and MEAH were both supercooled liquids after solvent removal in a rotary evaporator. During storage under reduced pressure for 3 days, both the PILs crystallized. It should be noted that after these PILs were crystallized, even when they were melted, they were prone to crystallization again also in the presence of small quantities of water. Preparation of liquid samples containing pure MEAA and MEAH was attempted with simultaneous melting and mixing, but even then, crystallization was randomly initiated in the liquid.

### Preparation of PIL–H_2_O Mixtures

During preparation of the PIL–H_2_O binary mixtures, ultrapure Milli-Q water was added to the PILs by syringe or micropipet with an accuracy of 2%. All samples were prepared considering the initial moisture content of the purified PIL. The mixtures were prepared in glass vials under a N_2_ atmosphere to prevent an increase in the moisture content of the PILs due to ambient humidity. For each PIL, two further samples containing 0.1 and 0.5 M of the cation or anion substrate in water were additionally prepared for NMR measurements. The compositions of all samples containing DEAA, DEAH, MEAA, and MEAH and varying amounts of water are presented in Table S1.

### Density Measurements of PILs in Water

Density (*ρ*, g cm^–3^) was measured for all PIL–H_2_O mixtures with the mole fraction of PIL *(X*_IL_) varying from 0.09 to 0.98. Measurements were carried out using an oscillating tube density meter (DMA5000M, Anton Paar, Austria) at temperatures ranging from 20 to 60 °C. The density meter was calibrated using air and Milli-Q water at 20 °C. The measurement accuracy was ±5 × 10^–5^ g cm^–3^. Additionally, density measurements were conducted for pure DEAA and DEAH, which contained only the original moisture content. For MEAA and MEAH, measurements began at a higher water content, over 1000 ppm, due to their crystalline form at 298 K, as mentioned above.

### Viscosity Measurements of PILs in Water

Viscosity was measured for PIL–H_2_O mixtures in a range of *X*_IL_ values from 0.09 to 0.98 using an Anton Paar rheometer MCR 72 at temperatures ranging from 20 to 60 °C. The uncertainty of the measurements was determined to be 4% using commercial viscosity standards. Similar to density measurements, viscosities of MEAA and MEAH were measured starting from a water content of at least 1000 ppm.

### Electrical Conductivity Measurements of PILs in Water

Electrical conductivities of MEAA, MEAH, DEAA, and DEAH were determined within the following *X*_IL_ ranges: 0.09–0.66, 0.09–0.59, 0.14–0.99, and 0.11–0.80, respectively. Measurements were conducted by using a Mettler Toledo InLab 731 conductivity electrode connected to a SevenEasy Benchtop Meter, calibrated with a 0.10 M KCl solution. Measurements were done at 25, 30, and 40 °C with a systematic uncertainty of 7% and at 50, 60, and 65 °C with a systematic uncertainty of 15%.

### ^1^H 1D and 2D NMR Spectroscopy

Nuclear magnetic resonance (NMR) data was acquired using an Agilent DD2 Spectrometer operating at 11.7 T (^1^H frequency = 500 MHz) and equipped with an inverse broadband probe. All spectra were processed using a MestReNova V14.3.2. The spectral assignment and labeling patterns for all cations and anions forming the PILs are presented in Figure 1.

Standard single-pulse ^1^H NMR spectra were acquired with a relaxation delay of 25 s and eight scans. ^1^H longitudinal relaxation (*T*_1_) was measured by using the inversion recovery pulse sequence with 10 values of the recovery delay selected between 0.06 and 32 s. ^1^H–^1^H Nuclear Overhauser Effect Spectroscopy (NOESY) 2D experiments were performed using four mixing times: MT = 400, 500, 600, and 700 ms. All measurements were done at 298 K (room temperature) and repeated at 60 °C, the latter with an additional 20 min of equilibration time applied after sample insertion to the spectrometer and prior to acquisition. Dipolar couplings were determined based on the intensity of the extracted cross- and diagonal peaks, following the procedure described elsewhere.^53^

### Critical Aggregation Concentration

The critical aggregation concentration (CAC) was determined for each sample using the method of continuous variation^54^ based on the density, viscosity, electrical conductivity, and ^1^H longitudinal relaxation times. The measured or calculated values of each variable were plotted as a function of the mole fraction of the IL (*X*_IL_), and the regression model with segmented relationship was applied to determine a single break point using the segmented package.^55^ The procedure was repeated at 20, 25, 30, 40, 50, and 60 °C, depending on the variable. The R script used to carry out the analysis can be found in Supporting Appendix S1.

## Results and Discussion

### Density of PILs in Water

Density (*ρ*) is a physical property in ionic liquids generally determined as relatively insensitive to the presence of water contamination.^56^ Herein, we aim to verify this claim for the subclass of PILs by determining changes in *ρ* as a function of the water content. The impact of intentionally added H_2_O molecules to four alkanolammonium- and carboxylic acid-based solvents is investigated at temperatures varying from 20 to 60 °C. PILs with ethanolammonium and diethanolammonium cations are selected to verify the differences between PILs with primary and secondary alkanolammonium cations. The selection of acetate and hexanoate anions allows the possible influence of the length of the alkyl chain in the anion.

Values of *ρ*_MEAA_ (**2A**), *ρ*_MEAH_ (**2B**), *ρ*_DEAA_ (**2C**), and *ρ*_DEAH_ (**2D**) measured at 20 °C as a function of the mole fraction of IL in the PIL–H_2_O binary mixtures (*X*_IL_) are presented in Figure 2. For comparison, the results previously published by Augusto et al.,^23^ Bressan et al.,^26^ and Santos et al.^57^ are added. All measured densities are available in Table S2.

**Figure 2:**
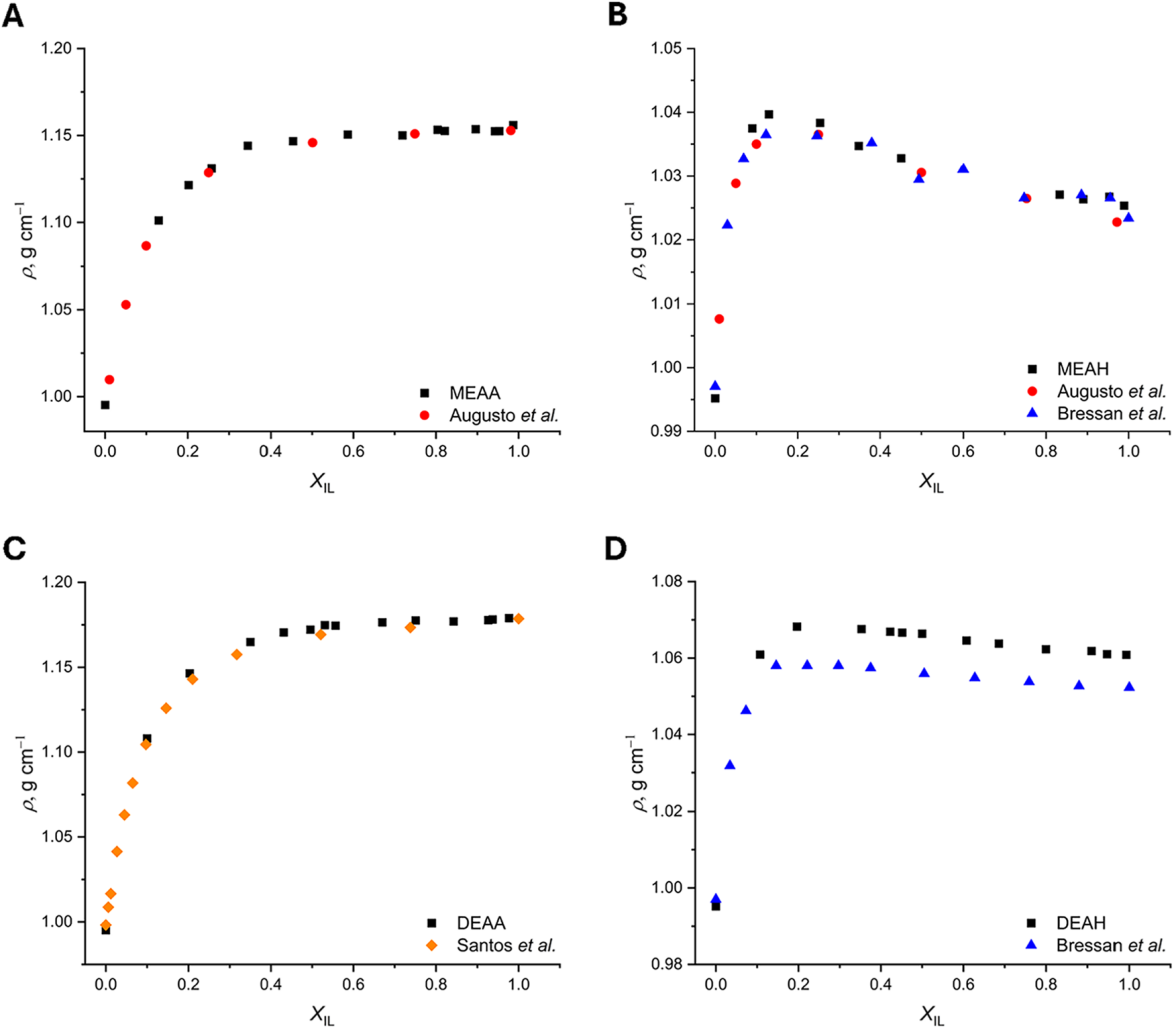
Densities (*ρ*) of four protic ionic liquids (PILs) measured in PIL–H_2_O binary mixtures at 20 °C and presented as a function of mole fraction of the ionic liquid (*X*_IL_): MEAA (A), MEAH (B), DEAA (C), and DEAH (D). The experimental results presented in this work are depicted with black squares. For comparison, the literature values for MEAA adapted from Augusto et al.^23^ (red dots), MEAH adapted from Augusto et al.^23^ and Bressan et al.^26^ (blue triangles), DEAA adapted from Santos et al.^57^ (orange diamond), and DEAH adapted from Bressan et al.^26^ are added.

As can be seen in Figure 2, the values of all measured densities are in good agreement with those available in the literature. In the case of ρ_DEAH_, the observed average difference of approximately 0.01 g cm^–3^ (0.9%) between the values measured in this study and those in the paper by Bressan et al.^26^ is due to the difference in measurement temperature of 5 °C, which was higher in the referenced work.

As predicted by trends reported in the literature for various IL and PIL structures, the hexanoate PILs, DEAH and MEAH, generally have densities lower than those of the acetate PILs, DEAA and MEAA, even in water mixtures. This is due to the presence of a longer alkyl chain in the anion.^25^ A comparison of both acetates shows *ρ*_DEAA_ being higher than that of *ρ*_MEAA_. Thus, the influence of hydroxyl groups in the cation on increasing density was also observed.^26,27^ Furthermore, a similar effect is seen in *ρ*_MEAH_ and *ρ*_DEAH_. However, the densities of PILs with the same anion remain similar, and the difference between them is less than 0.05 g cm^–3^. Therefore, it can be said that packing efficiencies in these PILs are similar despite the monoethanolammonium cation being significantly smaller than the diethanolammonium cation.^24,58^

As the *X*_IL_ values vary, a different influence on the PILs is observed. It can be seen in Figure 2 that increasing water content to *X*_IL_ 0.40–1.00 only causes a negligible change in *ρ*_MEAA_ and *ρ*_DEAA_ (0.002 g cm^–3^). For MEAH, a similar plateau is observed, while DEAH shows a local maximum at *X*_IL_ between 0.15 and 0.20. The nonsystematic trend in the PIL having a larger cation could be associated with the formation of larger number of local voids in the rigid solvent network, which will be filled with small quantities of water, before the overall dilution effect occurs, leading to an overall density decrease.^23^ For the same two PILs, a rapid increase of *ρ*_MEAH_ and *ρ*_DEAH_ is observed at *X*_IL_ = 0.00–0.20, whereas the change in density in MEAA and DEAA has a more gradual character below *X*_IL_ = 0.40.

The trends observed for acetate and hexanoate PILs differ, where the effect of water seems to depend more on the anion. Hexanoate PIL–H_2_O mixtures have maximum densities at *X*_IL_ = 0.13–0.20. When *X*_IL_ > 0.20, the value of *ρ* decreases. Similar observations have been reported for dibutylammonium (DBA) acetate, DBA propanoate, and DBA butanoate PILs.^59^ Thus, the introduced water molecules occupy the empty spaces between the cation and anion in this type of ionic liquids, and interact with the PIL within a certain composition range,^59^ rather than forming isolated clusters.

1 $$V_{m}^{E}=\left(\frac{X_{\mathrm{PIL}}M_{\mathrm{PIL}}+X_{H2O}M_{H2O}}{\rho }\right)-\left(\frac{X_{\mathrm{PIL}}M_{\mathrm{PIL}}}{\rho_{\mathrm{PIL}}}+\frac{X_{H2O}M_{H2O}}{\rho_{H2O}}\right)$$

The assessment of interactions between the components in a binary mixture can also be performed by calculating the excess molar volumes (*V*_m_^E^) based on values of ρ. Herein, *V*_m_^E^ values are calculated using eq 1, where *X*_PIL_ and *X*_H_2_O_ are the mole fractions of PIL and water, respectively; and *M*_PIL_ and *M*_H_2_O_ are the molar masses of PIL and water. *ρ*, *ρ*_PIL_, and *ρ*_H_2_O_ are the densities of the binary solution measured, pure PIL (including the initial water impurity), and pure water. The obtained *V*_m_^E^ values at 20 °C, presented in Figure 3, are negative for almost all PIL–H_2_O mixtures, confirming a better geometric fit and stronger interactions between the two different components of the mixture than between the pure components.^56^ In addition, the similarity in calculated trends confirms that water molecules bind predominantly to functional groups, defined as hydrogen bonding sites such as COO^–^, NH_3_^+^, or OH.^23^ Positive *V*_m_^E^ of MEAA with smaller water contents can be caused by the density that was used as *ρ*_PIL_ in the calculation. Ideally, *ρ*_PIL_ should be the density of PIL, which does not contain water at all, but in the present work, the density measured for the least water-containing PIL was used. All calculated *V*_m_^E^ values with calculated uncertainties can be found in Table S3.

**Figure 3:**
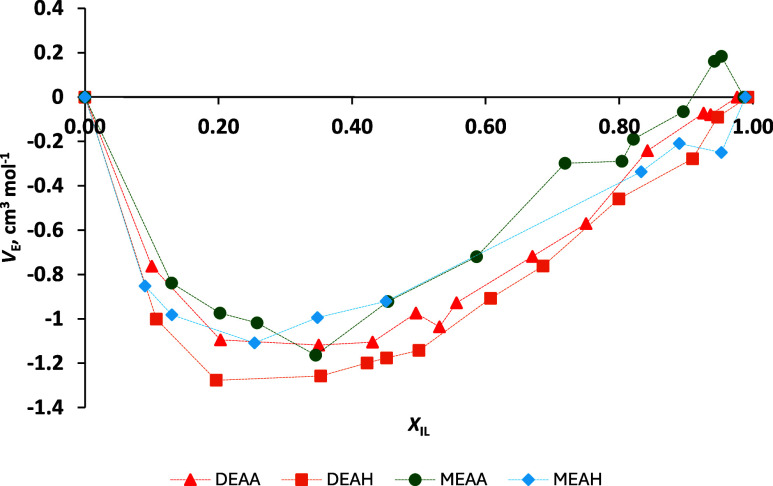
Excess molar volume (*V*_E_) of four protic ionic liquids (PILs) measured in a series of PIL–H_2_O binary mixtures with a varying mole fractions of the ionic liquid *X*_IL_ at 20 °C and presented as a function of mole fraction of ionic liquid *X*_IL_: DEAA (red triangle), DEAH (orange square), MEAA (green dot), and MEAH (blue diamond).

Densities of PIL–H_2_O mixtures were also measured at different temperatures (20, 30, 40, 50, and 60 °C), and the data can be found in Table S2. The density of all PIL–H_2_O mixtures decreases linearly in this temperature range, and the influence of the water content remains constant at the higher end of the range. Figure S1 illustrates an example of such a trend, which is additionally confirmed in the literature for PIL with similar structures.^56^

2 $$\alpha =-\frac{1}{\rho }\left(\frac{\Delta \rho }{\Delta T}\right)$$

The values of the density at different temperatures can be further used to calculate the isobaric thermal expansion coefficient, *α*, using eq 2. The density at temperature *T*_1_ is represented by *ρ*, and the differences in density and temperature are represented by Δ*ρ* and Δ*T*, respectively. Figure 4 shows the values of *α* for PIL–H_2_O mixtures at temperatures varying between 20 and 60 °C, and numerical data is reported in Table S4. Generally, a small change in the thermal expansion coefficient is observed in all PIL–H_2_O mixtures when the *X*_IL_ increases. The same was previously observed in pure PILs as well.^24,60^

**Figure 4:**
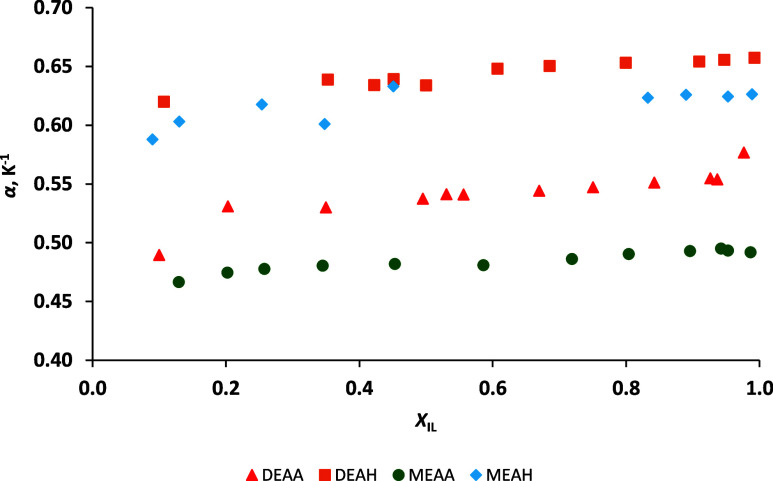
Comparison of thermal expansion coefficients *α* of four protic ionic liquids (PILs) measured in a series of PIL–H_2_O binary mixtures with a varying mole fraction of the ionic liquid *X*_IL_ at 20–60 °C and presented as a function of mole fraction of ionic liquid *X*_IL_: DEAA (red triangle), DEAH (orange square), MEAA (green dot), and MEAH (blue diamond).

The structures of cations and anions forming the PIL can also affect the thermal expansion (Figure 4). PILs containing a hexanoate anion have higher *α* values. In addition, between the two PILs with the same anion, those with a diethanolammonium cation have a higher *α*. Thus, PILs with longer or more aliphatic chains display weaker intermolecular associations, reduced by the steric repulsion as proposed in the work from Jackson et al.^61^ Furthermore, it can be seen in Figure 4 that adding more water to a PIL–H_2_O mixture slightly decreases *α*, indicating the existence of a higher number of hydrogen interactions in such mixtures. This increases the overall cohesiveness of the binary mixture and, therefore, also limits its thermal expansion.^59^

### Transport Properties of Protic Ionic Liquids in Water

Dynamic viscosity (*η*) and electrical conductivity (*κ*) are crucial transport properties, usually showing high sensitivity to changes in the water content in ILs.^62,63^ The viscosity of ionic liquids is highly affected by noncovalent interactions.^64,65^ Electrical conductivity is usually dependent on viscosity and helps to assess proton mobility in PILs and PIL–H_2_O mixtures.^66^ Therefore, measuring these properties, in addition to density analysis, could provide a deeper understanding of the role of the cation’s or anion’s structure in the intramolecular interactions in PIL–H_2_O mixtures.

In the present work, the values of *η*_DEAH_, *η*_DEAA_, *η*_MEAH_, and *η*_MEAA_ are measured at 20, 30, 40, 50, and 60 °C as a function of the mole fraction of IL (*X*_IL_) in PIL–H_2_O binary mixtures (Figure 5, numerical data in Table S5). Acetate PILs (left column) have higher viscosities than hexanoate PILs (right column). Moreover, the trend sequence of *η*_MEAA_ and *η*_DEAA_ reflects the sequence of ρ of these PILs: although it is not built from the largest cation and anion, DEAA has the highest density and viscosity among the studied PILs. Hence, it rather originates from the combination of a larger cation and the presence of additional hydroxyl groups that create more electrostatic interactions. However, Bressan et al. studied the densities and viscosities of mono-, di-, and triethanolammonium (TEA) hexanoate and found that MEA PIL has the lowest density and viscosity, and DEA PIL has the highest viscosity. In contrast, DEA PIL and TEA PIL have similar densities, with TEA slightly higher (0.02 g cm^–3^).^26^ For comparison, it has been shown that among PILs containing primary, secondary, and tertiary ammonium cations, substituted with only one OH group, *η* decreases as the number of organic groups attached to the nitrogen atom increases.^67^ Focusing on the role of anions, it has been reported that the viscosities of secondary alkylammonium cation and carboxylate PILs are higher when the alkyl chain of the anion is longer.^22^ PILs with a DEA cation in this work do not seem to follow this trend. Thus, these comparisons suggest that a complex balance exists between multiple types of noncovalent interactions, including the hydrogen bonds and the size and symmetry of ions.^68^ Consequently, the properties of specific ionic liquids should be investigated on a case-by-case basis.

**Figure 5:**
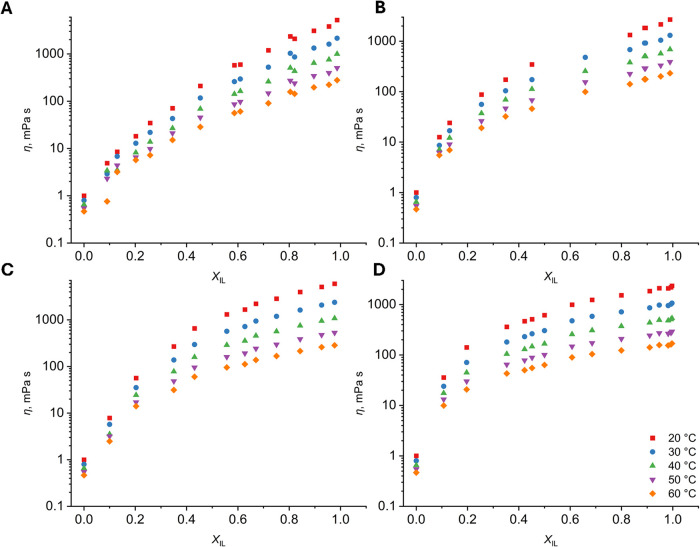
Viscosities (*η*) of four protic ionic liquids (PILs) measured in a series of PIL–H_2_O binary mixtures with a varying mole fraction of the ionic liquid *X*_IL_ at 20–60 °C (20 °C (red square), 30 °C (blue dot), 40 °C (green triangle), 50 °C (purple down-pointing triangle), and 60 °C (orange diamond)) and presented as a function of the mole fraction of ionic liquid *X*_IL_: MEAA (A), MEAH (B), DEAA (C), and DEAH (D). The *Y*-axis is shown on a logarithmic scale.

The dynamic viscosities of MEAA–H_2_O and MEAH–H_2_O mixtures, measured under similar conditions, are reported in the literature.^23^ For comparison, the results discussed herein and coming from Augusto et al., are compared in Figure S2. Data available in the literature was measured at 25 °C, whereas in the present study, 20 and 30 °C were used. Therefore, these results cannot be directly compared, but viscosity at 25 °C fits well between those at 20 and 30 °C, and the trends are comparable.

As presented in Figures 5 and S2, the addition of water has a strong influence on *η* of all PILs. A decrease of *X*_IL_ to 0.45 leads to *η*_MEAA_ and *η*_MEAH_ values changing by 95 and 87%, respectively, both at 20 °C in MEAA–H_2_O mixtures. This indicates that interactions in PIL are weakening due to increasing water content.^20^ A comparison of the curves in Figure 5 shows that the viscosity of mixtures where PILs have the same cation changes in a similar way. This is the opposite of the trends shown for density in Figure 2. This can be attributed to the smaller range of order and the more complicated structure of a PIL containing a diethanolammonium cation. Additionally, OH groups in the DEA cation form hydrogen bonds with water, thereby enhancing binding to the PIL structure. It has been reported that PILs containing an OH group on the ends of their alkyl chains have no or little polar and nonpolar segregation, which means that water would probably be homogeneously distributed throughout the PIL structure.^69^ This can explain the less steep slopes of the *X*_IL_ vs *η* trends for *η*_DEAA_ and *η*_DEAH_. Based on these findings, it seems that the cation has a greater influence on the structure of these PIL–H_2_O mixtures than the anion. However, the anion appears to have a more direct effect, as the viscosities of hexanoate PILs and PIL–H_2_O mixtures are lower than those of acetate PILs and PIL–H_2_O mixtures.

3 $$\eta_{E}=\eta -\left(\eta_{\mathrm{IL}}X_{\mathrm{IL}}+\eta_{H2O}X_{H2O}\right)$$

Strong interactions between PIL and water can also be confirmed by the viscosity deviation from the ideal behavior. The deviation of viscosity, i.e., excess viscosity (*η*_E_), was calculated by eq 3. Figure 6 presents the excess viscosities of PIL–H_2_O mixtures at 20 °C. Numerical values can be found in Table S6. Deviations for all mixtures are negative, indicating a decrease in the electrostatic interactions between ions due to the presence of water. Higher negative deviations were calculated for acetate PIL–H_2_O mixtures, which shows that an anion with a shorter alkyl chain leads to a higher number of interactions between ions and water. These results show that an anion has a greater influence on the strength of PIL–H_2_O interactions. In contrast, a cation exerts a greater influence on the incorporation of water within the PIL structure. It should be noted here, as a remark, that both MEA cation PILs crystallized at room temperature, which also indicates the influence of a cation on the PIL structure. Interestingly, MEAA–H_2_O and MEAH–H_2_O mixtures were able to crystallize even after the addition of approximately 25 mol % water.

**Figure 6:**
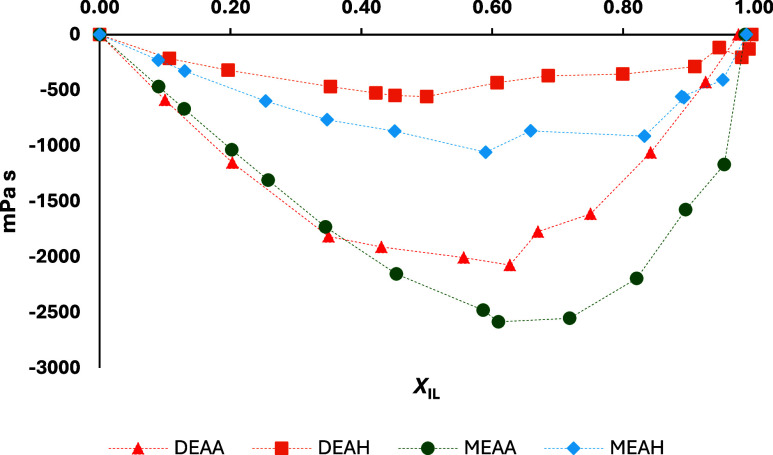
Excess viscosities (*η*_E_) of four PILs measured in a series of PIL–H_2_O binary mixtures with a varying mole fraction of the ionic liquid *X*_IL_ at 20 °C and presented as a function of mole fraction of ionic liquid *X*_IL_: DEAA (red triangle), DEAH (orange square), MEAA (green dot), and MEAH (blue diamond).

Electrical conductivity (*κ*) is dependent on viscosity and gives complementary information about the structure of the PIL–H_2_O mixtures, as well as the interactions between the mixture components. The electrical conductivities of different PIL–H_2_O mixtures, *κ*_DEAH_, *κ*_DEAA_, *κ*_MEAH_, and *κ*_MEAA_, at 25, 30, 40, 50, 60, and 65 °C are given in Table S7. As expected, the results show that the conductivity of all mixtures increases with temperature, likely due to decreased viscosity and higher diffusivity.^70^ Figure 7 shows the conductivities of the mixtures as a function of *X*_IL_ at 25 °C. The *κ* of PIL–H_2_O mixtures increases as the water content rises. This behavior is similar to that observed in previous studies, in which the peak conductivity of PIL–H_2_O mixtures was found to occur at a PIL mole fraction between 0.07 and 0.14.^56,69^ Therefore, the mixtures investigated in this study can also be described as being similar to concentrated salt solutions in water. In these solutions, conductivity increases rapidly in regions rich in water and decreases linearly with the mole fraction of the PIL in regions rich in salt.^69^ The magnitude of the conductivity increase is dependent on the PIL in the mixture. Acetate PIL–H_2_O mixtures have higher conductivities than hexanoate PIL–H_2_O mixtures, indicating better ion mobility in mixtures where the anion alkyl chain is shorter. Similar observations have also been made regarding other PILs based on alkyl- or alkanolammonium cations and carboxylate anions.^20,45^ This confirms that the interactions between water and ions are weaker than those between ions in more hydrophobic hexanoate PILs.

**Figure 7:**
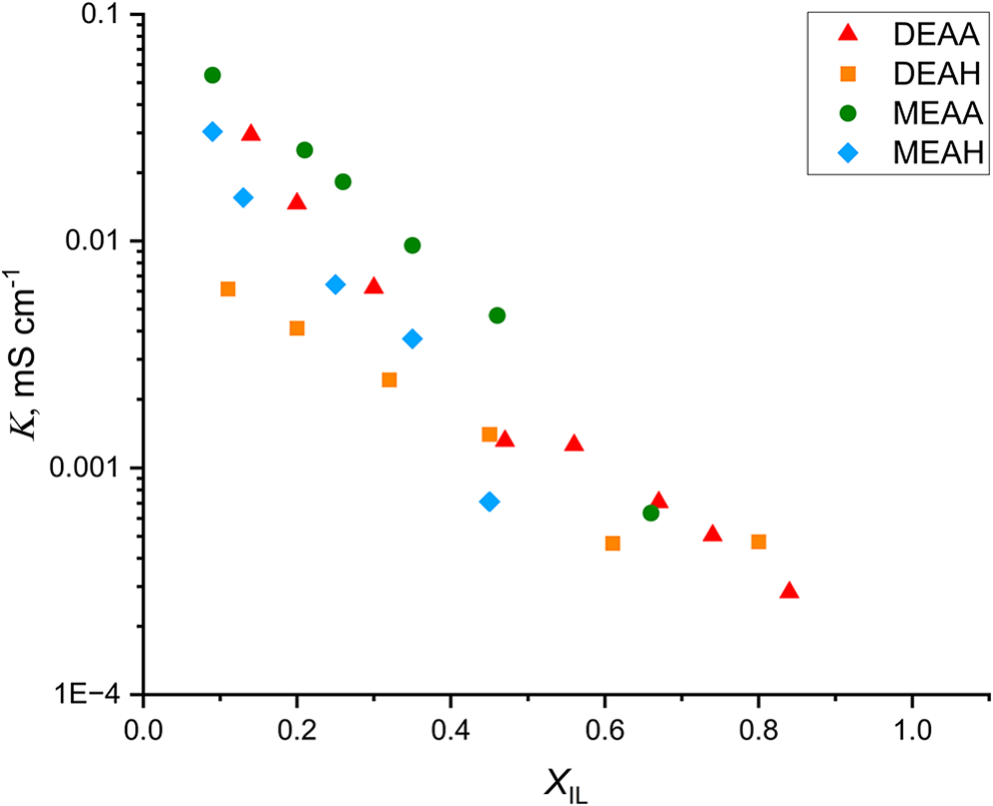
Electrical conductivities (*κ*) of four protic ionic liquids (PILs) measured in a series of PIL–H_2_O binary mixtures with a varying mole fraction of ionic liquid *X*_IL_ at 25 °C, presented as a function of the mole fraction of ionic liquid *X*_IL_: DEAA (red triangle), DEAH (orange square), MEAA (green dot), and MEAH (blue diamond).

### NMR Analysis

Due to the versatile nature of NMR spectroscopy, it has successfully paved its way to analyze the structure–property relationship in nonaqueous solvents, such as ILs or deep eutectic solvents.^46^ Figure 8 shows a comparison of seven ^1^H 1D NMR spectra acquired in DEAH–H_2_O binary mixtures with the *X*_IL_ varying between 0.12 and 1.0, the latter being a pure PIL. Similar figures for other PILs are available in Figures S6–S8. A systematic shielding of resonance assigned to NH_2_ and OH functional groups and water can be observed as *X*_IL_ decreases. This implies that from the beginning of the titration, the added H_2_O molecules are located in the vicinity of the functional groups, allowing fast proton exchange, as no isolated H_2_O resonance is detected.^44^ Interestingly, at *X*_IL_ = 1, when no water is added, the functional groups in both the cation and anion still remain represented by a single resonance, confirming their relative arrangement with respect to each other, allowing the fast exchange. The preferential alignment of hydroxyl groups in nonaqueous solvents is commonly observed in ILs or DESs consisting of multiple hydroxyl groups in both forming components.^53^ A similar effect was previously seen in the ρ analysis. Furthermore, migration of this peak from the initial chemical shift (CS) of 7.29 ppm, resulting from the combined influence of oxygen and nitrogen atoms, to 4.80 ppm, which remains close to the CS of pure water, reveals a significant shift in proton exchange kinetics. A similar trend is observed in all other PILs, with no distinct effect of the cation or anion on the occurrence of proton exchange between water molecules and the functional groups.

**Figure 8:**
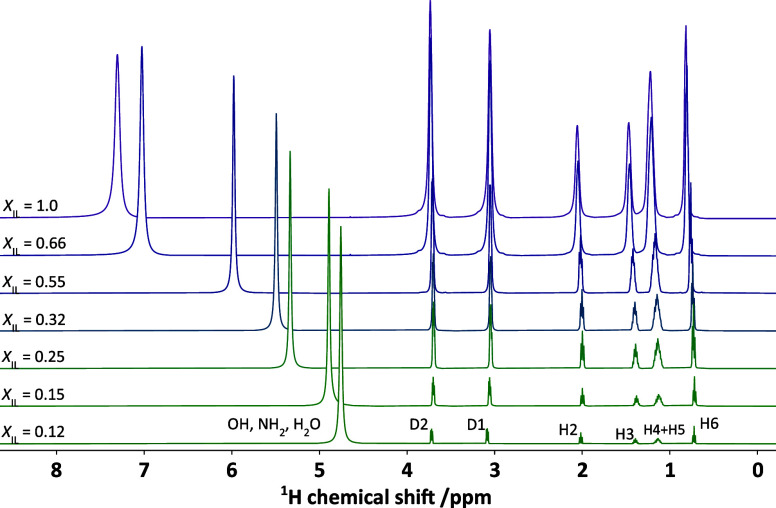
^1^H 1D NMR spectra were acquired at 298 K in a series of DEAH–H_2_O binary mixtures. The mole fraction of ionic liquid *X*_IL_ varies between 0.12 and 1.0, with the latter representing pure DEAH.

Because the CS of aliphatic protons are considered insensitive to changes in the solvent environment, ^1^H longitudinal (*T*_1_) relaxation times were additionally determined at 25 and 60 °C to understand the effect of water on the PIL cations and anions (numerical data is given in Table S8). Figure 9 shows a comparison of *T*_1_ values acquired in MEAH–H_2_O binary mixtures as a function of *X*_IL_. An analogous analysis is available in the Supporting Information for DEAA, DEAH, and MEAA. Overall, the values of *T*_1_ behave similarly in all PILs, showing two distinct regions above *X*_IL_ = 0.6 and below *X*_IL_ = 0.05. Between these two regions, the effect of water is more gradual in MEAA and MEAH. Interestingly, at a high temperature of 60 °C, a more abrupt change in proton relaxation is observed with no shift in the number of water molecules required to cause the *T*_1_ effect.

**Figure 9:**
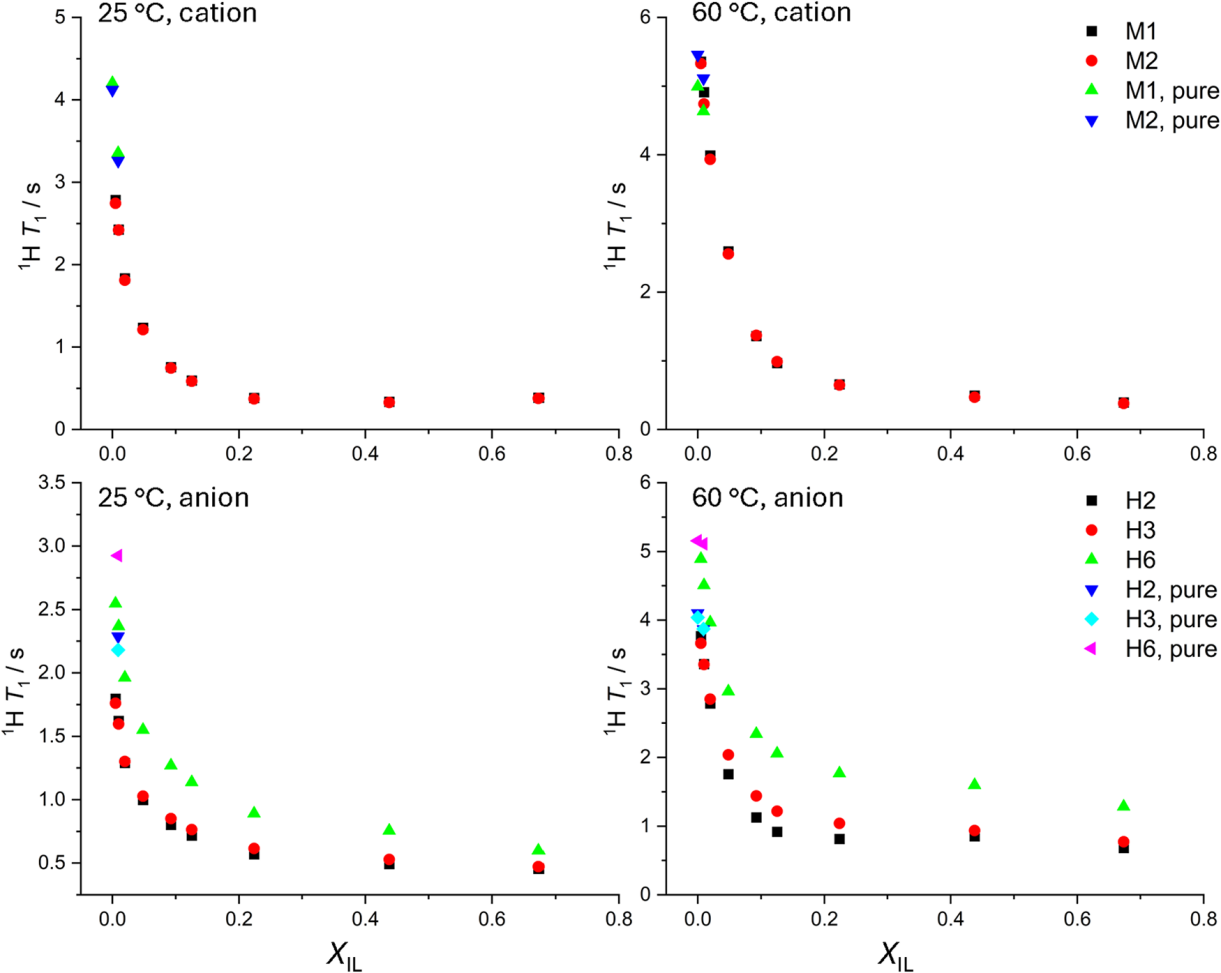
^1^H *T*_1_ (s) determined in monoethanolammonium hexanoate (MEAH)–water mixtures and represented as a function of the mole fraction of the ionic liquid *X*_IL_ for the cation (top) and anion (bottom). The ^1^H *T*_1_ values were measured at 25 °C (left) and 60 °C (right). Additional data (blue and green for the cation, light and dark blue, pink, all labeled as pure) present the ^1^H *T*_1_ of protons in the cation or anion separately in the single-compound solution. The labeling scheme is presented in Figure 1.

To support the electrical conductivity analysis, *T*_1_ values of protons in all used cations and anions were determined in pure 0.1 and 0.5 M solutions without the presence of the substrate for the counterion. Although the addition of water to solvents with a rigid structural arrangement, such as ionic liquids, is known to improve their conductivity, the excess of neutral water molecules will lead to a decrease of *κ* due to the formation of a percolating water network, isolating the IL cation and anion, defined as the only charge carriers.^44^ In Figure 9, the *T*_1_ of pure cation and anion remain 20–25% higher than those in the most diluted MEAH–H_2_O solutions. A similar effect is observed in three other PILs (Figures S3–S5). This confirms that the percolated water network was not yet formed, and even at relatively low *X*_IL_ < 0.05, the anion and cation still affect each other in the solution. At 60 °C, DEAH remains the only PIL in which the *T*_1_ of protons in ions in pure solutions or PIL–H_2_O mixtures still do not equilibrate at lowest *X*_IL_, due to the largest size of both, cation and anion, requiring more water molecules to reach full isolation.

Lastly, the magnitude of through-space inter- or intramolecular dipolar coupling σ (s^–1^) between the pairs of selected protons was calculated based on the relative intensity of cross-peaks detected in ^1^H–^1^H 2D NOESY experiments (see Figures S9 and S10 for examples). Because the reciprocal of σ is proportional to the distance between the two protons participating in the coupling, *r* ^6^ = $\frac{1}{\sigma }$ ,^53^ the values in Figure 10A, tabularized in Figure 10B, can be used to discuss the structural arrangement of cation and anion molecules in the DEAH–H_2_O binary mixtures. An analogous assessment of σ in MEAH–H_2_O is presented in Figure S11. In both cases, interactions of functional groups were excluded due to (I) a single peak corresponding to more than one type of proton in both the cation and anion and thus difficult to deconvolute the magnitude of its through-space interactions confidently and (II) the ongoing chemical exchange, which became the dominant process detected in 2D NMR, confirmed with 2D ROESY experiments.

**Figure 10:**
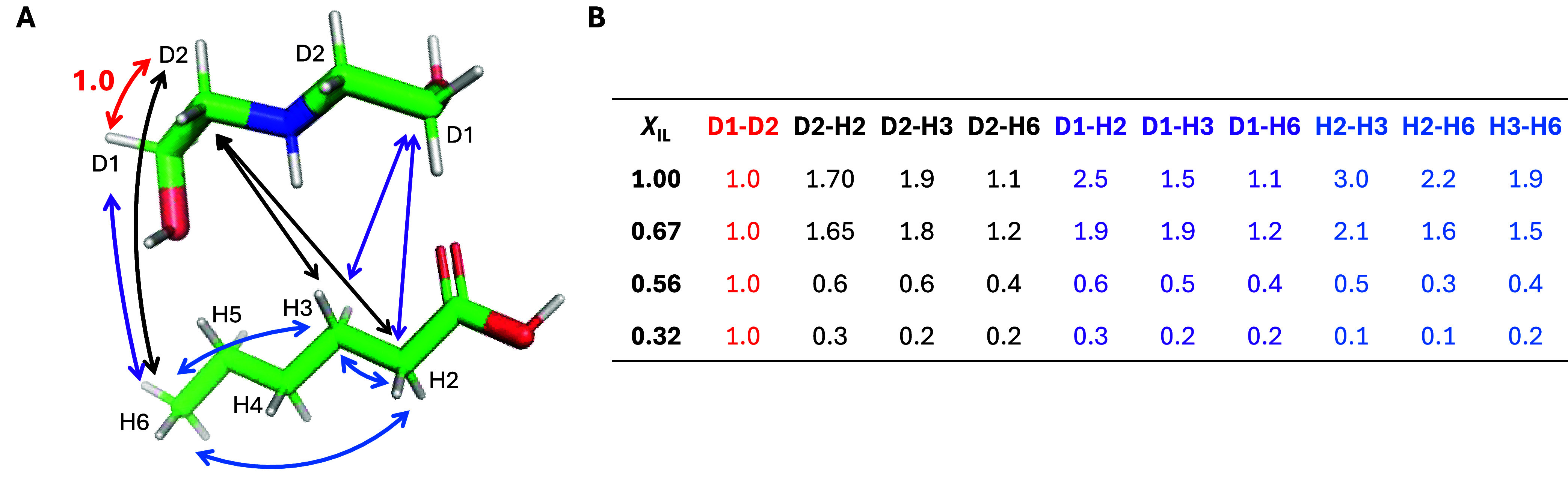
Dipolar couplings (σ, s^–1^) determined at 298 K in diethanolammonium hexanoate (DEAH)–water binary mixtures with a varying mole fraction of ionic liquid (IL) *X*_IL_. (A) Visual representation of all dipolar couplings determined between the cation (DEA) and anion (H) molecules and normalized for the intramolecular D1–D2 interaction. (B) Tabularized values of all dipolar couplings were calculated based on the relative intensity of cross-peaks detected in ^1^H–^1^H 2D NOESY experiments and are presented in panel (A) at four mole fractions of IL:*X*_IL_ = 1.0 (pure IL), 0.67, 0.56, and 0.32. Red: reference D1–D2 interaction; black: interactions of D1 in the cation; purple: interactions of D2 in the cation; blue: inter- and intramolecular interactions between protons in the anion.

In pure DEAH, the largest σ values are observed for the intermolecular D1–H2 pair, confirming the orientation of the functional group in the hexanoate anion toward the void between OH and NH_2_ groups in the diethanolammonium cation. Interestingly, the H2–H3 and H2–H6 interactions are respectively three and two times stronger than for D1–D2, implying that not only intra- but also intermolecular anion–anion interactions occur between the alkyl chains in the neighboring anions. Thus, due to the prevailing number of functional groups in the cation, aliphatic chains in the anion seem to be aligned together, similar to the patterns confirmed in imidazolium-based ILs with long alkyl side chains.^71^ This observation is further confirmed by the values for D2–H3 and D2–H6 being significantly smaller than those of all three H–H pairs.

When DEAH is doped with small quantities of water, at *X*_IL_ = 0.67, corresponding to a 1:2 H_2_O:PIL mole ratio, the interactions between H2–H3 and H2–H6 become most affected. On the contrary, D2–H2 and D2–H6 remain stable. Thus, water molecules are situated between the functional group and H2 in the anion and are more oriented toward the hydroxyl and not the amino functional groups in the cation. At higher water fractions, *X*_IL_ = 0.56 and 0.32, corresponding to roughly 1:1 and 2:1 H_2_O:PIL mole ratios, no selectivity in water distribution is observed, and all intermolecular interactions become weak. MEAH, the PIL with the same anion but a significantly smaller cation, exhibits a reverse trend (Figure S11). The addition of water increases the interactions between hexanoate anions, and also the aliphatic protons in the cation and anion, to a smaller degree. Functional groups on smaller MEA cations with significantly shorter chains are more easily accessible from the outer side than those interacting with the anion molecules.

Overall, NMR analysis allows us to hypothesize on the structural arrangement in DEAH and MEAH, in which the cation and anion are oriented toward each other with the functional groups and the hydrophobic alkyl chains in the anion form an environment similar to nonpolar domains confirmed in imidazolium ILs.^71^ This may also be the reason these PILs have lower electrical conductivity despite their lower viscosity. The introduced water molecules target the vicinity of the hydroxyl groups in the cation and disturb the cation–anion pairs from the inside (DEAH) or outside (MEAH). This is additionally confirmed with CAC analysis, in which twice as many water molecules are determined to disrupt the IL network (7–8:1 and 15:1 H_2_O:PIL mole ratio, respectively (Table S9)), despite the significantly smaller size of the anion.

### CAC Analysis

The method of continuous variation is most frequently applied to determine the H_2_O:IL mole ratio in ILs, at which the self-aggregation of the IL begins, and systematic, e.g., micelles and reversed micelles, or random aggregates are formed.^54,72^ At higher IL concentrations, they rearrange into a characteristic IL solvent network, and water molecules become isolated as single, paired molecules or in small clusters.^49^

In this study, the CAC was determined using the values of physicochemical properties (Figures 11, S12 and Table S10), such as *ρ*, *η*, *κ*, and also *T*_1_ NMR (Table S9), the last one giving an insight into the structural arrangement of cations and anions at varying water content. By performing a comprehensive analysis of four selected PILs, the possible influence of cation and/or anion size on the structural arrangement in the IL network will be verified, thereby confirming the existence of a structure–property relationship.

**Figure 11:**
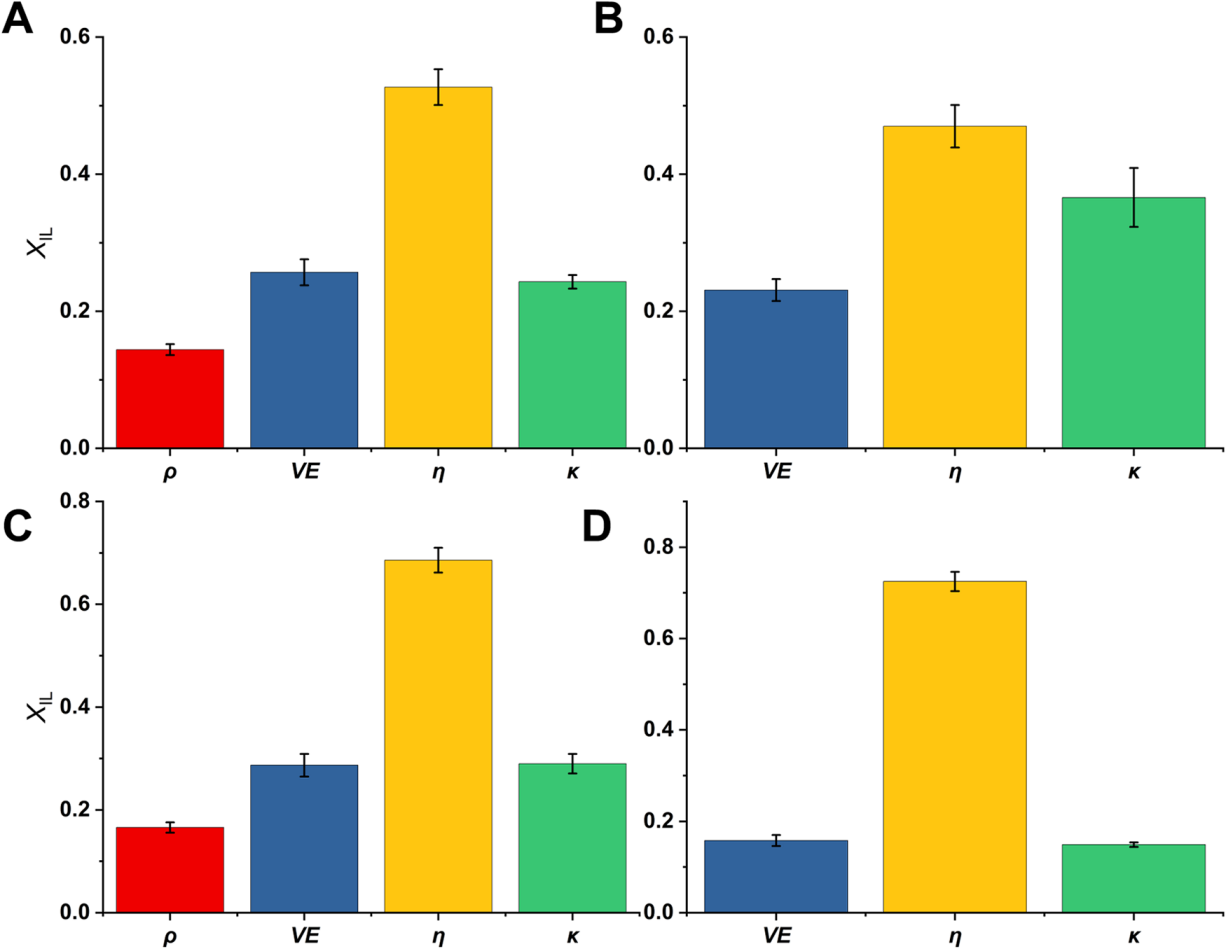
Critical aggregation concentrations of four protic ionic liquids (PILs), diethanolammonium acetate (DEAA, (A)), diethanolammonium hexanoate (DEAH, (B)), monoethanolammonium acetate (MEAA, (C)), and monoethanolammonium hexanoate (MEAH, (D)), in PIL–H_2_O binary mixtures with compositions corresponding to the mole fraction of the ionic liquid (*X*_IL_) determined using the density (*ρ*, red), excess volume (V_m_^E^, blue), viscosity (*η*, yellow), and conductivity (*κ*, green) measured at 20 °C. The numeric data is available in Table S10.

First, the values of CAC are determined using ρ as a function of *X*_IL_ for all PILs at a temperature range of 20–60 °C. The results of this analysis are presented in Table S10 and show that at 20 °C, the CAC for *ρ*_MEAA_ is at *X*_IL_ = 0.17 and for *ρ*_DEAA_ at *X*_IL_ = 0.14, which correspond to comparable H_2_O/PIL mole ratios of 5.0 and 6.0. These values align with the work of Thoppil et al. and Han et al., in which the higher hydrophobicity of the ions was confirmed to promote the aggregation of ILs in aqueous solutions.^73,74^ CAC analysis for *ρ*_MEAH_ and *ρ*_DEAH_ was not performed due to the linear character of the data. Thus, this confirms no significant breaking points in the PIL–H_2_O interactions.

Although *V*_m_^E^ is determined based on *ρ*, their CAC assessment shows different quantities of PIL needed to reach the critical aggregation. For the MEAH–H_2_O and DEAH–H_2_O binary mixtures, values corresponding to *X*_IL_ = 0.16 and *X*_IL_ = 0.23 are obtained, matching the values of maximum density. The CAC calculated for MEAA–H_2_O and DEAA–H_2_O binary mixtures correspond to *X*_IL_ values of 0.29 and 0.26. These values are consistent with minimum *V*_m_^E^ values found in the literature for similar PIL–H_2_O mixtures.^23,26,29,56^ Overall, MEAA and DEAA require a similar number of H_2_O molecules introduced per PIL ionic pair (2.5 and 3.0, respectively) to break down the solvent network. The latter, three H_2_O molecules per one PIL were determined in DEAH, while significantly more, five water molecules are required in MEAH to disrupt the solvent network.

CAC determined in all four PILs based on the *η* values suggests even fewer water molecules are needed to significantly change the viscosity behavior during the water-based titration. *η*_MEAA_ and *η*_MEAH_ begin to drastically change when the H_2_O/PIL ratio is approximately 0.5, while for *η*_DEAA_ and *η*_DEAH_ the ratio is around 1.0. CAC determined based on the conductivities shows no clear trend when comparing different cations and anions. For example, at 25 °C, *κ*_MEAH_ has a lowest CAC at *X*_IL_ = 0.15 and a H_2_O/PIL ratio of 6.0. This is consistent with previous studies, which indicate that the CAC decreases with increasing carbon atom number in the alkyl chain of the anion.^23,45^ However, the highest CAC was found for *κ*_DEAH_, assuming DEAH to consist of the two largest ions, corresponding to the H_2_O/PIL ratio of 2.0. The ratios for *κ*_DEAA_ and *κ*_MEAA_ are 3.0 and 2.5, respectively. Thus, it can be seen that the cation and anion provide separate trends in CAC determination.

To assess the final structure–property relationship in the investigated PILs, CAC determined using the trends in physicochemical properties is compared with the values obtained with NMR spectroscopy (Table S9). This analysis shows high reliability because CAC can be estimated separately for each proton in both cations and anions. Interestingly, the values of the estimated CACs based on *ρ*, *η*, and *κ* not only exhibit highly isolated cation- and/or anion-dependent trends but also do not align with NMR analysis.

DEAA, a PIL based on the acetate anion and larger of the alkanolammonium cations, reveals no coherent behavior for both alkali protons in the cation (C1 and C2) and anion. This implies a nonsystematic water distribution, prioritizing the formation of hydrogen bonds between functional groups and H_2_O molecules rather than a regular solvation behavior. In MEAA, and also DEAH and MEAH, all protons behave similarly in the cation and anion, and thus, CAC can be estimated.

The CAC determined in MEAA using NMR spectroscopy remains in agreement with *ρ* analysis. All other physicochemical properties are affected by water in significantly smaller quantities. Similarly, DEAH and MEAH show the highest number of water molecules needed to fully break down the PIL solvent network (8.0 and 15.0 H_2_O molecules per PIL, respectively). However, *η* and *κ* have their CAC determined to be below 5.0 water molecules per one PIL in both solvents and for all values. Thus, the number of water molecules accommodated by the solvent network cannot be used as a direct indicator of the shift in the physicochemical properties. On the other hand, a strong structure–property relationship is confirmed, in which each parameter must be evaluated separately and requires different amount of water to achieve the most prominent results while maintaining the overall PIL structure.

## Summary and Conclusions

Structure–property relationships, derived from the solvent structure and its interaction with water, were investigated in four alkanolammonium- and carboxylate-based protic ionic liquids (PILs), as well as their binary mixtures with water. The physicochemical and transport properties were measured, along with one- and two-dimensional nuclear magnetic resonance (NMR) spectroscopy, to assess the influence of water on the structural arrangement, verify hydrogen-bond formation, and determine the critical aggregation concentration for each PIL. It was observed that although the formation of hydrogen bonds remains the primary component in H_2_O–ionic liquid structural rearrangements in binary solutions, the size and symmetry of ions remain key factors governing the solvent properties. A summary of all observed trends is visualized in Figure 12. The critical aggregation concentration varied for different properties.

**Figure 12:**
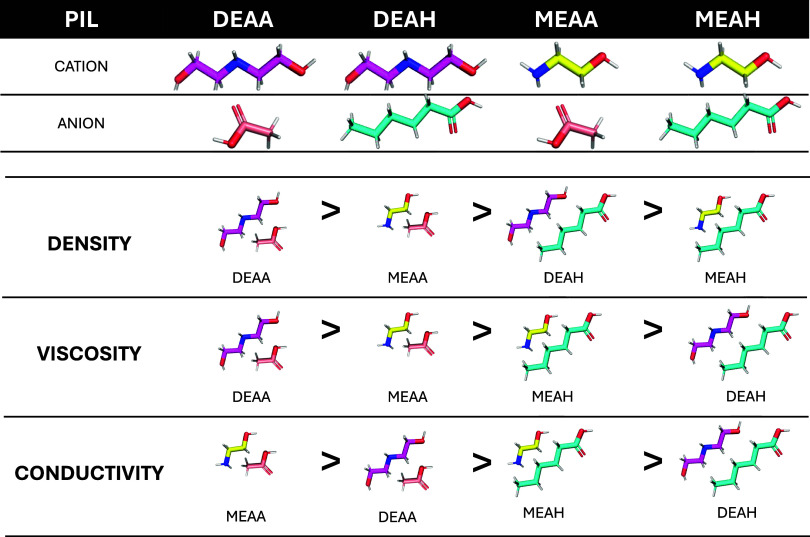
Visualization of the main trends determined in this study. Carbon atoms in the cation and anion substrates are presented with different colors (pink: monoethanolamine, MEA (M); yellow: diethanolamine, DEA (D); rose pink: acetic acid (A); and blue: hexanoic acid (H)). The color codes of oxygen (red), nitrogen (navy), and hydrogen (white) are consistent for all molecules.

The present study provides valuable input for property prediction models and molecular modeling of these types of protic ionic liquids. All PILs undergo a single-step breakdown of the solvent network at water contents comparable to those reported in the existing literature and other classes of ILs. However, their physicochemical properties are affected at significantly lower fractions of water, unlike those in other classes of ionic solvents. The high number of functional groups governs the solvent network. However, the properties remain influenced by the sizes and shapes of cations and anions. Consequently, there is a need to study not only PILs on a case-by-case basis but also the structure–property relationships established in the most studied classes of ILs, applying them to PILs and other less understood classes of ILs.

## Supplementary Material



## Notes

Overview of samples used in the study (Table S1); density data for all samples (Table S2); calculated excess molar volumes of all samples (Table S3); thermal expansion coefficients of all samples (Table S4); viscosity data for all samples (Table S5); calculated excess viscosity of all samples (Table S6); electrical conductivity data for all samples (Table S7); NMR longitudinal relaxation times of all samples (Table S8); critical aggregation concentration determined for all samples using ^1^H longitudinal relaxation time (Table S9); critical aggregation concentration determined for all samples using physicochemical properties (Table S10); density of DEAA–H_2_O mixtures presented as a function of temperature (Figure S1); viscosities of MEAA–H_2_O and MEAH–H_2_O mixtures compared with literature data (Figure S2); ^1^H longitudinal relaxation time determined in DEAA–H_2_O mixtures as a function of ionic liquid mole fraction (Figure S3); ^1^H longitudinal relaxation time determined in DEAH–H_2_O mixtures as a function of ionic liquid mole fraction (Figure S4); ^1^H longitudinal relaxation time determined in MEAA–H_2_O mixtures as a function of iosnic liquid mole fraction (Figure S5); ^1^H 1D NMR spectra for DEAA–H_2_O mixtures (Figure S6); ^1^H 1D NMR spectra for MEAA–H_2_O mixtures (Figure S7); ^1^H 1D NMR spectra for MEAH–H_2_O mixtures (Figure S8); ^1^H 2D NOESY spectrum of pure DEAH (Figure S9); ^1^H 2D NOESY spectrum of pure MEAH (Figure S10); dipolar couplings of MEAA–H_2_O mixtures (Figure S11); example of the application of method of continuous variation (Figure S12); and R script for the estimation of critical aggregation concentrations (Appendix S1) (PDF)
