# Supplementary material for: Protic Ionic Liquid–H2O Mixtures—Structure, Interactions, and Structure–Property Relationships
Source: J Phys Chem B. 2026 Jan 27;130(5):1661–74. doi: 10.1021/acs.jpcb.5c07863 (PMC12884516; doi:10.1021/acs.jpcb.5c07863)
Supplement: Supplementary file 1 [file jp5c07863_si_001.pdf]

Protic Ionic Liquid-H_2O Mixtures - Structure, Interactions and Structure-Property Relationships

Liisa-Maria Kaljusmaa[a,*], Katarzyna Maria Dziubinska-Kuehn[b], Balázs Erdős[c] Diandra Tubli[a], Sander Lillepea[a], Indrek Reile[b], and Oliver Järvik[a]

January 8, 2026

[a] *Tallinn University of Technology, Ehitajate tee 5, 19086, Tallinn, Estonia*
[b] *National Institute of Chemical Physics and Biophysics, Akadeemia Tee 23, 12618, Tallinn, Estonia.*
[c] *Department of Data Science and Knowledge Discovery, Simula Metropolitan Center for Digital Engineering, Stensberggata 27, 0170 Oslo, Norway.*
[*] *Contact person: Liisa-Maria Kaljusmaa, liisamaria.kaljusmaa@taltech.ee*

Table S1: Overview of samples used in the study, sorted by the protic ionic liquid (PIL) and the mole fraction of the ionic liquid (X_{IL}) in the PIL-H_2O binary mixtures.

X_{IL}, DEAA			
Density, ρ	Viscosity, η	Conductivity, κ	NMR, δ
0.10	0.10	0.14	0.005
0.20	0.20	0.20	0.01
0.35	0.35	0.30	0.02
0.43	0.43	0.47	0.05
0.50	0.56	0.56	0.09
0.53	0.63	0.67	0.13
0.56	0.67	0.74	0.26
0.67	0.75	0.84	0.50
0.75	0.84	0.99	0.69
0.84	0.93		0.99
0.93	0.98		
0.94			
0.98			
X_{IL}, DEAH			
Density, ρ	Viscosity, η	Conductivity, κ	NMR, δ
0.11	0.11	0.11	0.001
0.20	0.20	0.20	0.002
0.35	0.35	0.32	0.039
0.42	0.42	0.45	0.014
0.45	0.45	0.61	0.009
0.50	0.50	0.80	0.005
0.61	0.61		0.093
0.69	0.69		0.116
0.80	0.80		0.147
0.91	0.91		0.247
0.95	0.95		0.323
0.99	0.98		0.551
	0.993		0.657
	0.997		1.000
X_{IL}, MEAA			
Density, ρ	Viscosity, η	Conductivity, κ	NMR, δ
0.13	0.09	0.09	0.0099
0.20	0.13	0.21	0.01
0.26	0.20	0.26	0.02
0.35	0.26	0.35	0.05
0.45	0.35	0.46	0.09
0.59	0.45	0.66	0.13
0.72	0.59		0.22
0.80	0.61		0.47
0.82	0.72		0.69
0.90	0.82		0.99
0.94	0.90		
0.95	0.95		
0.99	0.99		

X_{IL}, MEAH			
Density, ρ	Viscosity, η	Conductivity, κ	NMR, δ
0.09	0.09	0.09	0.005
0.13	0.13	0.13	0.01
0.25	0.25	0.25	0.02
0.35	0.35	0.35	0.05
0.45	0.45	0.45	0.09
0.59	0.59	0.59	0.13
0.66	0.66		0.22
0.83	0.83		0.44
0.89	0.89		0.67
0.95	0.89		0.99
0.99	0.95		
	0.99		

Table S2: The densities (ρ, g cm^{-3}) of four protic ionic liquids (PILs), diethanolammonium acetate (DEAA), diethanolammonium hexanoate (DEAH), monoethanolammonium acetate (MEAA), monoethanolammonium hexanoate (MEAH), measured in PIL-H_2O binary mixtures with compositions corresponding to PIL molar fractions (X_{IL}).

DEAA

T, °C	20	30	40	50	60
X_{IL}	g cm^{-3}				
0.00	0.9952	0.9957	0.9922	0.9880	0.9833
0.10	1.1081	1.1031	1.0978	1.0923	1.0866
0.20	1.1464	1.1406	1.1347	1.1286	1.1223
0.35	1.1649	1.1589	1.1529	1.1467	1.1404
0.43	1.1705	1.1645	1.1583	1.1521	1.1494
0.50	1.1722	1.1660	1.1599	1.1536	1.1472
0.53	1.1749	1.1686	1.1624	1.1561	1.1497
0.56	1.1745	1.1683	1.1621	1.1558	1.1494
0.67	1.1765	1.1703	1.1639	1.1576	1.1512
0.75	1.1776	1.1713	1.1649	1.1585	1.1521
0.84	1.1770	1.1707	1.1642	1.1578	1.1514
0.93	1.1778	1.1714	1.1650	1.1584	1.1519
0.94	1.1781	1.1718	1.1654	1.1588	1.1523
0.98	1.1790	1.1723	1.1654	1.1587	1.1521

DEAH

T, °C	20	30	40	50	60
X_{IL}	g cm^{-3}				
0.00	0.9952	0.9957	0.9922	0.9880	0.9833
0.11	1.0609	1.0548	1.0484	1.0419	1.0349
0.20	1.0683	1.0617	1.0551	1.0414	1.0483
0.35	1.0676	1.0610	1.0543	1.0475	1.0407
0.42	1.0669	1.0604	1.0538	1.0470	1.0402
0.45	1.0667	1.0601	1.0534	1.0466	1.0397
0.50	1.0664	1.0599	1.0532	1.0465	1.0397
0.61	1.0646	1.0578	1.0511	1.0443	1.0374
0.69	1.0638	1.0570	1.0502	1.0434	1.0365
0.80	1.0623	1.0556	1.0487	1.0419	1.0349
0.91	1.0618	1.0551	1.0482	1.0414	1.0344
0.95	1.0610	1.0542	1.0473	1.0405	1.0336
0.99	1.0609	1.0540	1.0471	1.0402	1.0333

**MEAA**					
T, °C	20	30	40	50	60
X_{IL}	g cm^{-3}				
0.00	0.9952	0.9957	0.9922	0.9880	0.9833
0.13	1.1011	1.0964	1.0914	1.0862	1.0807
0.20	1.1215	1.1164	1.1112	1.1059	1.1004
0.26	1.1310	1.1259	1.1206	1.1152	1.1096
0.35	1.1441	1.1387	1.1333	1.1278	1.1223
0.45	1.1467	1.1413	1.1359	1.1304	1.1248
0.59	1.1505	1.1452	1.1397	1.1342	1.1286
0.72	1.1501	1.1445	1.1391	1.1335	1.1279
0.80	1.1533	1.1477	1.1421	1.1365	1.1309
0.82	1.1526	1.1483	1.1429	1.1374	1.1318
0.90	1.1536	1.1480	1.1423	1.1367	1.1311
0.94	1.1524	1.1469	1.1413	1.1354	1.1298
0.95	1.1525	1.1464	1.1413	1.1356	1.1300
0.99	1.1560	1.1504	1.1448	1.1391	1.1335

**MEAH**					
T, °C	20	30	40	50	60
X_{IL}	g cm^{-3}				
0.00	0.9952	0.9957	0.9922	0.9880	0.9833
0.09	1.0375	1.0318	1.0259	1.0197	1.0134
0.13	1.0397	1.0337	1.0276	1.0213	1.0149
0.25	1.0383	1.0322	1.0259	1.0195	1.0130
0.35	1.0347	1.0293	1.0230	1.0166	1.0101
0.45	1.0328	1.0265	1.0201	1.0136	1.0069
0.59	1.0306	1.0244	1.0181	1.0117	1.0052
0.66	1.0294	1.0232	1.0169	1.0106	1.0042
0.83	1.0271	1.0208	1.0145	1.0082	1.0018
0.89	1.0264	1.0201	1.0137	1.0074	1.0010
0.95	1.0268	1.0205	1.0141	1.0078	1.0014
0.99	1.0254	1.0191	1.0128	1.0064	1.0000

Table S3: The excess molar volumes (V_m^E, cm^3 mol^{-1}) and their uncertainties (unc.) of four protic ionic liquids (PILs), diethanolammonium acetate (DEAA), diethanolammonium hexanoate (DEAH), monoethanolammonium acetate (MEAA), monoethanolammonium hexanoate (MEAH), measured in PIL-H_2O binary mixtures with compositions corresponding to PIL molar fractions (X_{IL}).

T, °C	20		30		40		50		60	
**DEAA**										
X_{IL}	V_m^E	Unc.	V_m^E	Unc.	V_m^E	Unc.	V_m^E	Unc.	V_m^E	Unc.
0.1	-0.7623	0.0381	-0.7012	0.0351	-0.7000	0.0350	-0.7011	0.0351	-0.7021	0.0351
0.20	-1.0949	0.0547	-1.0391	0.0520	-1.0395	0.0520	-1.0376	0.0519	-1.0364	0.0518
0.35	-1.1183	0.0559	-1.0879	0.0544	-1.1042	0.0552	-1.1168	0.0558	-1.1258	0.0563
0.43	-1.1050	0.0553	-1.0863	0.0543	-1.1102	0.0555	-1.1251	0.0563	-1.3582	0.0679
0.50	-0.9737	0.0487	-0.9594	0.0480	-0.9905	0.0495	-1.0088	0.0504	-1.0180	0.0509
0.53	-1.0361	0.0518	-1.0226	0.0511	-1.0540	0.0527	-1.0748	0.0537	-1.0830	0.0541
0.56	-0.9278	0.0464	-0.9173	0.0459	-0.9532	0.0477	-0.9745	0.0487	-0.9826	0.0491
0.67	-0.7188	0.0359	-0.7277	0.0364	-0.7613	0.0381	-0.7899	0.0395	-0.7996	0.0400
0.75	-0.5713	0.0286	-0.5895	0.0295	-0.6252	0.0313	-0.6542	0.0327	-0.6650	0.0332
0.84	-0.2421	0.0121	-0.2688	0.0134	-0.3053	0.0153	-0.3327	0.0166	-0.3414	0.0171
0.93	-0.0724	0.0036	-0.1059	0.0053	-0.1538	0.0077	-0.1684	0.0084	-0.1763	0.0088
0.94	-0.0797	0.0040	-0.1162	0.0058	-0.1669	0.0083	-0.1819	0.0091	-0.1916	0.0096
**DEAH**										
X_{IL}	V_m^E	Unc.	V_m^E	Unc.	V_m^E	Unc.	V_m^E	Unc.	V_m^E	Unc.
0.11	-1.0016	0.0501	-0.9205	0.0460	-0.8975	0.0449	-0.8771	0.0439	-0.8506	0.0425
0.20	-1.2773	0.0639	-1.2005	0.0600	-1.1823	0.0591	-0.7991	0.0400	-1.5126	0.0756
0.35	-1.2582	0.0629	-1.2081	0.0604	-1.2041	0.0602	-1.1952	0.0598	-1.1858	0.0593
0.42	-1.1990	0.0599	-1.1681	0.0584	-1.1765	0.0588	-1.1791	0.0590	-1.1716	0.0586
0.45	-1.1761	0.0588	-1.1419	0.0571	-1.1489	0.0574	-1.1444	0.0572	-1.1398	0.0570
0.50	-1.1422	0.0571	-1.1222	0.0561	-1.1400	0.0570	-1.1477	0.0574	-1.1490	0.0575
0.61	-0.9072	0.0454	-0.8746	0.0437	-0.8926	0.0446	-0.8910	0.0445	-0.8785	0.0439
0.69	-0.7621	0.0381	-0.7319	0.0366	-0.7515	0.0376	-0.7535	0.0377	-0.7424	0.0371
0.80	-0.4590	0.0230	-0.4486	0.0224	-0.4569	0.0228	-0.4611	0.0231	-0.4487	0.0224
0.91	-0.2778	0.0139	-0.2777	0.0139	-0.2883	0.0144	-0.2974	0.0149	-0.2871	0.0144
0.95	-0.0904	0.0045	-0.0907	0.0045	-0.0985	0.0049	-0.1046	0.0052	-0.0925	0.0046

MEAA

T, °C	20		30		40		50		60	
X_{IL}	V_m^E	Unc.	V_m^E	Unc.	V_m^E	Unc.	V_m^E	Unc.	V_m^E	Unc.
0.13	-0.8383	0.0419	-0.7734	0.0387	-0.7660	0.0383	-0.7643	0.0382	-0.7619	0.0381
0.20	-0.9743	0.0487	-0.9134	0.0457	-0.9060	0.0453	-0.9057	0.0453	-0.9060	0.0453
0.26	-1.0180	0.0509	-0.9606	0.0480	-0.9542	0.0477	-0.9556	0.0478	-0.9567	0.0478
0.35	-1.1640	0.0582	-1.1132	0.0557	-1.1089	0.0554	-1.1134	0.0557	-1.1169	0.0558
0.45	-0.9227	0.0461	-0.8826	0.0441	-0.8793	0.0440	-0.8846	0.0442	-0.8873	0.0444
0.59	-0.7200	0.0360	-0.6945	0.0347	-0.6968	0.0348	-0.7043	0.0352	-0.7068	0.0353
0.72	-0.2991	0.0150	-0.2726	0.0136	-0.2768	0.0138	-0.2843	0.0142	-0.2845	0.0142
0.80	-0.2893	0.0145	-0.2724	0.0136	-0.2649	0.0132	-0.2749	0.0137	-0.2731	0.0137
0.82	-0.1905	0.0095	-0.2715	0.0136	-0.2835	0.0142	-0.2987	0.0149	-0.2994	0.0150
0.90	-0.0655	0.0033	-0.0542	0.0027	-0.0442	0.0022	-0.0514	0.0026	-0.0483	0.0024
0.94	0.1612	-0.0081	0.1685	-0.0084	0.1720	-0.0086	0.1841	-0.0092	0.1856	-0.0093
0.95	0.1843	-0.0092	0.2374	-0.0119	0.1915	-0.0096	0.2028	-0.0101	0.2001	-0.0100

MEAH

X_{IL}	V_m^E	Unc.	V_m^E	Unc.	V_m^E	Unc.	V_m^E	Unc.	V_m^E	Unc.
0.09	-0.8522	0.0426	-0.7672	0.0384	-0.7420	0.0371	-0.7230	0.0361	-0.7043	0.0352
0.13	-0.9821	0.0491	-0.8982	0.0449	-0.8722	0.0436	-0.8528	0.0426	-0.8335	0.0417
0.25	-1.1085	0.0554	-1.0343	0.0517	-1.0107	0.0505	-0.9950	0.0498	-0.9774	0.0489
0.35	-0.9939	0.0497	-0.9848	0.0492	-0.9645	0.0482	-0.9520	0.0476	-0.9360	0.0468
0.45	-0.9211	0.0461	-0.8644	0.0432	-0.8399	0.0420	-0.8232	0.0412	-0.7883	0.0394
0.59	-0.7772	0.0389	-0.7394	0.0370	-0.7295	0.0365	-0.7243	0.0362	-0.7076	0.0354
0.66	-0.6556	0.0328	-0.6197	0.0310	-0.6212	0.0311	-0.6297	0.0315	-0.6247	0.0312
0.83	-0.3374	0.0169	-0.3212	0.0161	-0.3082	0.0154	-0.3236	0.0162	-0.3234	0.0162
0.89	-0.2098	0.0105	-0.1965	0.0098	-0.1816	0.0091	-0.1922	0.0096	-0.1923	0.0096
0.95	-0.2498	0.0125	-0.2516	0.0126	-0.2327	0.0116	-0.2573	0.0129	-0.2585	0.0129

Table S4: The thermal expansion coefficients (α) of four protic ionic liquids (PILs), diethanolammonium acetate (DEAA), diethanolammonium hexanoate (DEAH), monoethanolammonium acetate (MEAA), monoethanolammonium hexanoate (MEAH), in PIL-H_2O binary mixtures with compositions corresponding to PIL molar fractions (X_{IL}), calculated in the temperature range between 20 ° and 60 °C.

DEAA

X_{IL}	Average ρ	$\Delta\rho$	α
	g cm^{-1}	g cm^{-1}	K^{-1}
0.00	0.9909	0.0119	0.000300
0.10	1.0976	0.0215	0.00049
0.20	1.1345	0.0241	0.000531
0.35	1.1528	0.0244	0.000530
0.43	1.1590	0.0211	0.000456
0.50	1.1598	0.0249	0.000537
0.53	1.1623	0.0252	0.000541
0.56	1.1620	0.0252	0.000541
0.67	1.1639	0.0253	0.000544
0.75	1.1649	0.0255	0.000547
0.84	1.1642	0.0257	0.000551
0.93	1.1649	0.0258	0.000555
0.94	1.1653	0.0258	0.000554
0.98	1.1655	0.0269	0.000577

DEAH

X_{IL}	Average ρ	$\Delta\rho$	α
	g cm^{-1}	g cm^{-1}	K^{-1}
0.00	0.9909	0.0119	0.000300
0.11	1.0482	0.0260	0.000620
0.20	1.0549	0.0199	0.000473
0.35	1.0542	0.0269	0.000639
0.42	1.0536	0.0267	0.000634
0.45	1.0533	0.0269	0.000639
0.50	1.0531	0.0267	0.000634
0.61	1.0510	0.0273	0.000648
0.69	1.0502	0.0273	0.000650
0.80	1.0487	0.0274	0.000653
0.91	1.0482	0.0274	0.000654
0.95	1.0473	0.0275	0.000656
0.99	1.0471	0.0275	0.000657

MEAA

X_{IL}	Average ρ	$\Delta\rho$	α
	g cm^{-1}	g cm^{-1}	K^{-1}
0.00	0.9909	0.0119	0.000300
0.13	1.0912	0.0204	0.000467
0.20	1.1111	0.0211	0.000475
0.26	1.1205	0.0214	0.000478
0.35	1.1332	0.0218	0.000480
0.45	1.1358	0.0219	0.000482
0.59	1.1397	0.0219	0.000481
0.72	1.1390	0.0221	0.000486
0.80	1.1421	0.0224	0.000490
0.82	1.1426	0.0208	0.000456
0.90	1.1424	0.0225	0.000493
0.94	1.1412	0.0226	0.000495
0.95	1.1412	0.0225	0.000493
0.99	1.1447	0.0225	0.000492

MEAH			
X_{IL}	Average ρ	$\Delta\rho$	α
	g cm^{-1}	g cm^{-1}	K^{-1}
0.00	0.9909	0.0119	0.000300
0.09	1.0256	0.0241	0.000588
0.13	1.0275	0.0248	0.000603
0.25	1.0258	0.0253	0.000618
0.35	1.0228	0.0246	0.000601
0.45	1.0200	0.0258	0.000633
0.59	1.0180	0.0254	0.000624
0.66	1.0169	0.0252	0.000619
0.83	1.0145	0.0253	0.000623
0.89	1.0137	0.0254	0.000626
0.95	1.0141	0.0253	0.000624
0.99	1.0127	0.0254	0.000626

Table S5: The viscosities (η) of four protic ionic liquids (PILs), diethanolammonium acetate (DEAA), diethanolammonium hexanoate (DEAH), monoethanolammonium acetate (MEAA), monoethanolammonium hexanoate (MEAH), measured in PIL-H_2O binary mixtures with compositions corresponding to PIL molar fractions (X_{IL}).

**DEAA**, η					
T, °C	20	30	40	50	60
X_{IL}	mPa s				
0.00	1.0	0.80	0.65	0.55	0.47
0.10	7.9	5.7	3.5	3.2	2.5
0.20	57	35	24	17	14
0.35	269	138	78	48	31
0.43	655	297	159	96	60
0.56	1308	569	288	160	96
0.63	1663	717	355	192	112
0.67	2219	942	457	242	139
0.74	2857	1188	566	296	167
0.84	3957	1618	753	386	214
0.93	5091	2070	946	476	260
0.98	5957	2379	1079	532	285
**DEAH,** η					
T, °C	20	30	40	50	60
X_{IL}	mPa s				
0.00	1.0	0.80	0.65	0.55	0.47
0.11	36	24	18	13	9.9
0.20	142	71	45	30	21
0.35	362	181	105	65	43
0.42	466	232	130	78	50
0.45	512	264	147	89	55
0.50	617	303	167	101	64
0.61	994	477	258	148	90
0.69	1240	585	307	172	104
0.80	1523	714	372	208	124
0.91	1848	863	437	244	142
0.95	2106	976	494	272	157
0.98	2099	954	483	265	155
0.993	2200	1017	510	277	163

MEAA, η

T, °C	20	30	40	50	60
X_{IL}	mPa s				
0.00	1.0	0.80	0.65	0.55	0.47
0.09	4.9	2.9	3.4	2.3	0.76
0.13	8.5	6.8	3.5	4.4	3.2
0.20	18	13	8.2	6.5	5.7
0.26	35	22	14	9.8	7.2
0.35	71	43	27	21	15
0.45	211	117	69	45	29
0.59	579	260	142	87	56
0.61	595	296	164	97	61
0.72	1198	524	260	148	91
0.82	2090	861	433	240	144
0.90	3098	1334	646	342	196
0.95	3812	1615	760	396	223
0.99	5217	2150	996	507	279

MEAH, η

T, °C	20	30	40	50	60
X_{IL}	mPa s				
0.00	1.0	0.80	0.65	0.55	0.47
0.09	13	8.7	7.1	6.2	5.5
0.13	24	17	12	9.1	7.0
0.25	88	56	37	26	19
0.35	174	104	70	47	32
0.45	347	174	112	68	46
0.59	533	293	175	112	74
0.66	912	480	256	155	99
0.83	1331	682	380	226	141
0.89	1842	918	500	292	177
0.89	1842	918	500	292	177
0.95	2162	1050	569	330	201
0.99	2697	1310	691	389	232

Table S6: The excess viscosities (η_E) of four protic ionic liquids (PILs), diethanolammonium acetate (DEAA), diethanolammonium hexanoate (DEAH), monoethanolammonium acetate (MEAA), monoethanolammonium hexanoate (MEAH), measured in PIL-H_2O binary mixtures with compositions corresponding to PIL molar fractions (X_{IL}).

DEAA

T, °C	20		30		40		50		60	
-	η_E	Unc.	η_E	Unc.	η_E	Unc.	η_E	Unc.	η_E	Unc.
X_{IL}	mPa s									
0.10	-589	-41	-233	-16	-105	-7	-50	-4	-26	-2
0.20	-1153	-81	-448	-31	-195	-14	-91	-6	-44	-3
0.35	-1816	-127	-695	-49	-300	-21	-138	-10	-69	-5
0.43	-1912	-134	-728	-51	-306	-21	-134	-9	-63	-4
0.56	-2007	-141	-755	-53	-312	-22	-136	-10	-63	-4
0.63	-2074	-145	-776	-54	-322	-23	-142	-10	-67	-5
0.67	-1773	-124	-652	-46	-265	-19	-114	-8	-53	-4
0.75	-1614	-113	-597	-42	-244	-17	-103	-7	-47	-3
0.84	-1061	-74	-386	-27	-156	-11	-63	-4	-27	-2
0.93	-427	-30	-133	-9	-53	-4	-17	-1	-5	-0.3

DEAH

T, °C	20		30		40		50		60	
-	η_E	Unc.	η_E	Unc.	η_E	Unc.	η_E	Unc.	η_E	Unc.
X_{IL}	mPa s									
0.11	-216	-15	-91	-6	-40	-3	-18	-1	-9	-1
0.20	-320	-22	-140	-10	-61	-4	-28	-2	-13	-1
0.35	-467	-33	-197	-14	-84	-6	-38	-3	-17	-1
0.42	-526	-37	-221	-15	-97	-7	-45	-3	-22	-2
0.45	-548	-38	-220	-15	-95	-7	-43	-3	-21	-1
0.50	-557	-39	-232	-16	-102	-7	-45	-3	-21	-1
0.61	-433	-30	-174	-12	-68	-5	-29	-2	-13	-1
0.69	-370	-26	-149	-10	-61	-4	-28	-2	-12	-1
0.80	-355	-25	-142	-10	-57	-4	-25	-2	-12	-1
0.91	-288	-20	-111	-8	-52	-4	-22	-2	-12	-1
0.95	-118	-8	-38	-3	-14	-1	-5	0	-3	-0.2
0.98	-206	-14	-97	-7	-44	-3	-22	-2	-11	-1
0.99	-131	-9	-45	-3	-23	-2	-13	-1	-5	-0.4

MEAA

T, °C	20		30		40		50		60	
-	η_E	Unc.	η_E	Unc.	η_E	Unc.	η_E	Unc.	η_E	Unc.
X_{IL}	mPa s									
0.09	-467	-33	-192	-13	-87	-6	-44	-3	-25	-2
0.13	-668	-47	-272	-19	-126	-9	-62	-4	-33	-2
0.20	-1037	-73	-422	-30	-194	-14	-96	-7	-51	-4
0.26	-1310	-92	-533	-37	-243	-17	-121	-8	-65	-5
0.35	-1731	-121	-700	-49	-318	-22	-154	-11	-82	-6
0.45	-2156	-151	-858	-60	-383	-27	-185	-13	-98	-7
0.59	-2480	-174	-1001	-70	-442	-31	-211	-15	-108	-8
0.61	-2585	-181	-1015	-71	-444	-31	-212	-15	-110	-8
0.72	-2552	-179	-1022	-72	-457	-32	-216	-15	-110	-8
0.82	-2194	-154	-904	-63	-385	-27	-176	-12	-86	-6
0.90	-1576	-110	-592	-41	-247	-17	-112	-8	-54	-4
0.95	-1169	-82	-438	-31	-191	-13	-88	-6	-43	-3

MEAH

T, °C	20		30		40		50		60	
-	η_E	Unc.	η_E	Unc.	η_E	Unc.	η_E	Unc.	η_E	Unc.
X_{IL}	mPa s									
0.09	-231	-16	-110	-8	-56	-4	-29	-2	-16	-1
0.13	-327	-23	-154	-11	-78	-5	-42	-3	-24	-2
0.25	-598	-42	-277	-19	-139	-10	-73	-5	-40	-3
0.35	-765	-54	-352	-25	-171	-12	-89	-6	-49	-3
0.45	-870	-61	-418	-29	-199	-14	-108	-8	-59	-4
0.59	-1059	-74	-480	-34	-233	-16	-118	-8	-63	-4
0.66	-865	-61	-383	-27	-200	-14	-102	-7	-54	-4
0.83	-915	-64	-409	-29	-195	-14	-99	-7	-52	-4
0.89	-567	-40	-253	-18	-117	-8	-56	-4	-31	-2
0.89	-558	-39	-248	-17	-115	-8	-54	-4	-30	-2
0.95	-408	-29	-199	-14	-90	-6	-41	-3	-20	-1

Table S7: The electrical conductivities (κ) of four protic ionic liquids (PILs), diethanolammonium acetate (DEAA), diethanolammonium hexanoate (DEAH), monoethanolammonium acetate (MEAA), monoethanolammonium hexanoate (MEAH), measured in PIL-H_2O binary mixtures with compositions corresponding to PIL molar fractions (X_{IL}).

DEAA

T, °C	25	30	40	50	60	65
-	κ					
X_{IL}	mS cm^{-1}					
0.14	25	29.4	35.2	41.3	46.9	49.5
0.2	12.31	14.64	19.02	23.8	28.8	30.9
0.3	4.47	6.22	9.08	12.11	15.34	16.93
0.47	0.95	1.32	2.27	3.6	5.03	6.19
0.56	0.9	1.26	2.24	3.5	5.03	5.98
0.67	0.45	0.71	1.34	2.14	3.24	3.78
0.74	0.37	0.51	0.94	1.63	2.55	3.22
0.84	0.15	0.28	0.57	1.05	1.65	2.02
0.99	0.13	0.21	0.43	0.78	1.27	1.58

DEAH

T, °C	25	30	40	50	60	65
-	κ					
X_{IL}	mS cm^{-1}					
0.11	4.87	6.14	8	9.88	11.89	13.07
0.2	3.11	4.12	5.51	7.08	8.76	9.66
0.32	1.8	2.44	3.65	4.89	6.38	7.2
0.45	0.99	1.41	2.1	2.93	3.91	4.51
0.61	0.35	0.47	0.76	1.18	1.64	1.84
0.8	0.34	0.47	0.79	1.21	1.75	2.11

MEAA

T, °C	25	30	40	50	60	65
-	κ					
X_{IL}	mS cm^{-1}					
0.09	46.3	53.9	64.2	72.3	79.4	82.7
0.21	20.4	25.2	32.4	39.7	46.5	50.2
0.26	14.91	18.26	24.4	30.3	36.2	38.9
0.35	6.87	9.56	13.89	18.25	22.9	25.2
0.46	3.39	4.69	7.29	10.18	13.49	14.94
0.66	0.6	0.63	1.13	3.72	5.82	7.03

MEAH

T, °C	25	30	40	50	60	65
-	κ					
X_{IL}	mS cm^{-1}					
0.09	24.7	28.8	34.6	39.8	44	46.3
0.13	19.06	22.1	26.8	31.3	35.3	37.2
0.25	5.73	7.21	9.75	12.1	14.76	15.73
0.35	2.55	3.47	4.85	6.42	7.95	8.67
0.45	1.125	1.561	2.25	3.15	4.3	4.71
0.59	0.505	0.668	1.078	1.656	2.31	2.68

Table S8: The NMR longitudinal relaxation time (T_1, s) of four protic ionic liquids (PILs), diethanolammonium acetate (DEAA), diethanolammonium hexanoate (DEAH), monoethanolammonium acetate (MEAA), monoethanolammonium hexanoate (MEAH), in PIL-H_2O binary mixtures with compositions corresponding to PIL molar fractions (X_{IL}) determined using the inversion recovery experiment in NMR spectroscopy. The NMR labeling pattern for all cations and anions is presented in Figure 1.

DEAA, 25 °C

**Label**	**D1**		**D2**		**A2**	
X_{IL}	T_1, s	error, s	T_1, s	error, s	T_1, s	error, s
1.00	1.09	0.003	1.10	0.001	1.12	0.006
0.69	0.76	0.006	0.76	0.004	0.84	0.021
0.50	0.52	0.006	0.53	0.004	0.74	0.032
0.26	0.34	0.002	0.33	0.002	0.81	0.009
0.13	0.40	0.001	0.36	0.001	1.08	0.012
0.09	0.50	0.005	0.45	0.005	1.57	0.013
0.05	0.78	0.013	0.68	0.014	2.36	0.017
0.02	1.19	0.017	1.05	0.016	3.11	0.033
0.01	1.64	0.005	1.32	0.010	3.49	0.033
0.005	2.29	0.007	1.76	0.006	3.55	0.031

.

DEAA, 60 °C

**Label**	**D1**		**D2**		**A2**	
X_{IL}	T_1, s	error, s	T_1, s	error, s	T_1, s	error, s
1.00	0.47	0.005	0.47	0.005	0.83	0.046
0.69	0.39	0.002	0.38	0.002	0.58	0.115
0.50	0.37	0.002	0.35	0.001	0.36	0.091
0.26	0.53	0.006	0.45	0.003	0.42	0.103
0.13	0.97	0.016	0.83	0.010	0.98	0.242
0.09	1.33	0.007	1.13	0.013	2.18	0.363
0.05	2.00	0.024	1.75	0.025	2.95	0.576
0.02	2.87	0.028	2.52	0.023	5.21	0.379
0.01	3.32	0.051	2.88	0.033	6.47	0.175
0.005	3.62	0.104	3.11	0.056	7.25	0.113

DEAH, 25 °C

Label	D1		D2		H2		H3		H6	
X_{IL}	T_1, s	error, s	T_1, s	error, s	T_1, s	error, s	T_1, s	error, s	T_1, s	error, s
1.000	0.64	0.008	0.65	0.006	0.69	0.004	0.69	0.001	0.77	0.021
0.657	0.65	0.003	0.65	0.004	0.67	0.002	0.66	0.002	0.80	0.030
0.551	0.54	0.005	0.54	0.005	0.59	0.002	0.58	0.001	0.72	0.023
0.323	0.41	0.005	0.40	0.005	0.60	0.006	0.61	0.005	0.83	0.022
0.247	0.37	0.004	0.36	0.004	0.62	0.004	0.62	0.003	0.90	0.016
0.147	0.38	0.001	0.35	0.001	0.69	0.001	0.67	0.001	1.10	0.002
0.116	0.41	0.001	0.37	0.000	0.72	0.001	0.72	0.001	1.17	0.004
0.093	0.46	0.002	0.41	0.003	0.76	0.001	0.77	0.001	1.26	0.005
0.005	0.68	0.011	0.61	0.012	0.89	0.007	0.92	0.006	1.51	0.007
0.009	1.14	0.011	1.01	0.017	1.25	0.013	1.25	0.014	1.93	0.004
0.014	1.28	0.011	1.13	0.018	1.36	0.014	1.35	0.015	2.03	0.004
0.039	1.45	0.012	1.27	0.018	1.49	0.020	1.46	0.020	2.15	0.013
0.002	1.67	0.020	1.46	0.023	1.66	0.039	1.63	0.043	2.27	0.040
0.001	1.47	0.108	1.41	0.070	1.52	0.103	1.58	0.046	2.24	0.047

DEAH, 60 °C

Label	D1		D2		H2		H3		H6	
X_{IL}	T_1, s	error, s	T_1, s	error, s	T_1, s	error, s	T_1, s	error, s	T_1, s	error, s
1.000	0.46	0.009	0.46	0.009	0.72	0.007	0.76	0.009	1.06	0.039
0.657	0.42	0.005	0.41	0.005	0.72	0.006	0.74	0.007	1.18	0.023
0.551	0.40	0.005	0.38	0.005	0.71	0.002	0.74	0.002	1.17	0.009
0.323	0.45	0.003	0.40	0.003	0.81	0.007	0.87	0.001	1.45	0.022
0.247	0.51	0.003	0.45	0.002	0.88	0.007	0.95	0.001	1.57	0.023
0.147	0.88	0.005	0.76	0.005	1.16	0.010	1.29	0.001	2.02	0.039
0.116	0.75	0.004	0.65	0.002	1.08	0.010	1.19	0.002	1.97	0.033
0.093	1.06	0.006	0.91	0.005	1.26	0.010	1.41	0.002	2.15	0.036
0.005	1.79	0.009	1.58	0.007	1.83	0.012	1.99	0.008	2.94	0.025
0.009	2.77	0.013	2.45	0.010	2.89	0.008	2.92	0.020	4.10	0.011

MEAA, 25 °C

Label	M1		M2		A2	
X_{IL}	T_1, s	error, s	T_1, s	error, s	T_1, s	error, s
0.67	0.29	0.001	0.28	0.001	0.46	0.017
0.50	0.43	0.001	0.42	0.001	0.63	0.073
0.25	0.75	0.005	0.72	0.003	0.81	0.175
0.13	1.12	0.009	1.07	0.012	1.11	0.256
0.09	1.66	0.005	1.61	0.005	2.43	0.214
0.05	2.24	0.003	2.21	0.004	3.45	0.162
0.02	2.45	0.016	2.51	0.007	3.95	0.126
0.01	2.61	0.011	2.65	0.005	4.30	0.047

MEAA, 60 °C

Label	M1		M2		A2	
X_{IL}	T_1, s	error, s	T_1, s	error, s	T_1, s	error, s
0.67	0.39	0.029	0.37	0.027	0.14	0.025
0.50	0.92	0.077	0.87	0.069	0.22	0.053
0.25	1.29	0.275	1.29	0.226	0.26	0.085
0.13	2.10	0.401	1.75	0.430	0.23	0.088
0.09	2.24	0.457	1.69	0.322	4.63	0.498
0.05	4.13	0.409	4.32	0.249	5.14	0.586
0.02	4.32	0.465	4.87	0.082	6.58	0.246
0.01	4.03	0.725	5.12	0.100	7.19	0.150

MEAH, 25 °C										
**Label**	**M1**		**M2**		**H2**		**H3**		**H6**	
X_{IL}	T_1, s	error, s	T_1, s	error, s	T_1, s	error, s	T_1, s	error, s	T_1, s	error, s
0.674	0.39	0.007	0.38	0.008	0.46	0.003	0.47	0.003	0.60	0.023
0.438	0.34	0.007	0.33	0.007	0.49	0.004	0.53	0.002	0.75	0.016
0.224	0.38	0.002	0.37	0.003	0.57	0.003	0.62	0.002	0.89	0.011
0.125	0.59	0.001	0.58	0.001	0.72	0.003	0.76	0.001	1.14	0.012
0.093	0.76	0.005	0.74	0.002	0.80	0.006	0.85	0.002	1.27	0.026
0.048	1.23	0.003	1.21	0.001	1.00	0.004	1.03	0.002	1.55	0.015
0.020	1.84	0.004	1.81	0.003	1.29	0.005	1.30	0.006	1.96	0.016
0.010	2.43	0.056	2.42	0.041	1.62	0.020	1.60	0.023	2.37	0.024
0.005	2.78	0.110	2.74	0.064	1.80	0.027	1.76	0.032	2.55	0.021

MEAH, 60 °C										
**Label**	**M1**		**M2**		**H2**		**H3**		**H6**	
X_{IL}	T_1, s	error, s	T_1, s	error, s	T_1, s	error, s	T_1, s	error, s	T_1, s	error, s
0.674	0.39	0.008	0.38	0.008	0.68	0.008	0.77	0.006	1.28	0.019
0.438	0.49	0.007	0.47	0.007	0.85	0.007	0.94	0.006	1.60	0.008
0.224	0.66	0.005	0.65	0.005	0.81	0.012	1.04	0.007	1.77	0.051
0.125	0.97	0.002	0.99	0.002	0.92	0.007	1.22	0.004	2.06	0.045
0.093	1.36	0.002	1.37	0.004	1.13	0.008	1.44	0.003	2.34	0.044
0.048	2.59	0.013	2.56	0.016	1.76	0.018	2.04	0.005	2.96	0.052
0.020	3.99	0.016	3.93	0.008	2.79	0.015	2.85	0.009	3.96	0.031
0.010	4.91	0.163	4.74	0.101	3.36	0.047	3.35	0.054	4.51	0.054
0.005	5.35	0.186	5.33	0.155	3.77	0.094	3.66	0.075	4.89	0.072

Table S9: The critical aggregation concentrations of four protic ionic liquids (PILs), diethanolammonium acetate (DEAA), diethanolammonium hexanoate (DEAH), monoethanolammonium acetate (MEAA), monoethanolammonium hexanoate (MEAH), in PIL-H_2O binary mixtures with compositions corresponding to PIL molar fractions (X_{IL}) determined using ^{1}H longitudinal (T_1) relaxation times in NMR spectroscopy. The NMR labeling pattern for all cations and anions is presented in Figure 1.

^{1}H T_1 at 25 °C

	DEAA			DEAH			MEAA			MEAH		
NMR label	X_{IL}	error	H_2O/PIL	X_{IL}	error	H_2O/PIL	X_{IL}	error	H_2O/PIL	X_{IL}	error	H_2O/PIL
C1	0.03	0.005	32.3	0.101	0.009	8.9	0.155	0.009	5.5	0.064	0.009	14.6
C2	0.062	0.01	15.1	0.1	0.009	9.0	0.151	0.006	5.6	0.064	0.009	14.6
A2	0.138	0.009	6.2	0.122	0.011	7.2	0.143	0.009	6.0	0.06	0.009	15.7
A3	-			0.124	0.01	7.1	-			0.06	0.009	15.7
A6	-			0.163	0.014	5.1	-			0.062	0.009	15.1

.

^{1}H T_1 at 60 °C

	DEAA			DEAH			MEAA			MEAH		
NMR label	X_{IL}	error	H_2O/PIL	X_{IL}	error	H_2O/PIL	X_{IL}	error	H_2O/PIL	X_{IL}	error	H_2O/PIL
C1	0.108	0.012	8.3	0.124	0.026	7.1	0.15	0.038	5.7	0.071	0.007	13.1
C2	0.137	0.014	6.3	0.123	0.025	7.1	0.095	0.013	9.5	0.097	0.01	9.3
A2	0.064	0.009	14.6	0.156	0.041	5.4	0.146	0.026	5.8	0.064	0.005	14.6
A3	-			0.125	0.03	7.0	-			0.067	0.006	13.9
A6	-			0.126	0.031	6.9	-			0.065	0.006	14.4

Table S10: The critical aggregation concentration (CAC) of four protic ionic liquids (PILs), diethanolammonium acetate (DEAA), diethanolammonium hexanoate (DEAH), monoethanolammonium acetate (MEAA), monoethanolammonium hexanoate (MEAH), in PIL-H_2O binary mixtures with compositions corresponding to PIL molar fractions (X_{IL}) determined from density, excess molar volume, and viscosity data. For each CAC value, the corresponding number of H_2O molecules per one PIL molecule in the binary mixture is calculated and presented in the last column. The density of MEAH and DEAS was excluded from the analysis due to the linear trend in the data.

DENSITY, ρ (g cm^{-3})

-	DEAA			DEAH			MEAA			MEAH		
T, °C textitX$_{\mathrm{IL}}$	X_{IL} error	error H_2O/PIL	H_2O/PIL X_{IL}	X_{IL} error	error H_2O/PIL	H_2O/PIL						
20	0.144	0.008	5.9		-		0.166	0.01	5		-	
30	0.215	0.017	3.7		-		0.168	0.011	5		-	
40	0.146	0.009	5.8		-		0.168	0.011	5		-	
50	0.216	0.017	3.6		-		0.168	0.011	5		-	
60	0.147	0.01	5.8		-		0.168	0.011	5		-	

EXCESS VOLUME, V_E^m (cm^3 mol^{-1})

-	DEAA			DEAH			MEAA			MEAH		
T, °C	X_{IL}	error	H_2O/PIL	X_{IL}	error	H_2O/PIL	X_{IL}	error	H_2O/PIL	X_{IL}	error	H_2O/PIL
20	0.257	0.019	2.9	0.231	0.016	3.3	0.287	0.022	2.5	0.158	0.012	5.3
30	0.264	0.02	2.8	0.238	0.016	3.2	0.293	0.024	2.4	0.164	0.013	5.1
40	0.268	0.021	2.7	0.242	0.017	3.1	0.294	0.024	2.4	0.166	0.013	5.0
50	0.271	0.022	2.7	0.374	0.033	1.7	0.295	0.024	2.4	0.166	0.014	5.0
60	0.282	0.026	2.5	0.208	0.01	3.8	0.296	0.024	2.4	0.166	0.014	5.0

VISCOSITY, η (mPa s)

-	DEAA			DEAH			MEAA			MEAH		
T, °C	X_{IL}	error	H_2O/PIL	X_{IL}	error	H_2O/PIL	X_{IL}	error	H_2O/PIL	X_{IL}	error	H_2O/PIL
20	0.527	0.026	0.9	0.47	0.031	1.1	0.686	0.024	0.5	0.725	0.021	0.4
30	0.522	0.025	0.9	0.468	0.0034	1.1	0.686	0.024	0.5	0.768	0.034	0.3
40	0.515	0.026	0.9	0.432	0.037	1.3	0.683	0.024	0.5	0.758	0.031	0.3
50	0.509	0.027	1.0	0.408	0.045	1.5	0.677	0.025	0.5	0.749	0.034	0.3
60	0.501	0.029	1.0	0.425	0.042	1.4	0.668	0.025	0.5	0.572	0.046	0.7

EXCESS VISCOSITY, η_E (mPA s)

-	DEAA			DEAH			MEAA			MEAH		
T, °C	X_{IL}	error	H_2O/PIL	X_{IL}	error	H_2O/PIL	X_{IL}	error	H_2O/PIL	X_{IL}	error	H_2O/PIL
20	0.523	0.029	0.9	0.465	0.025	1.2	0.674	0.015	0.5	0.558	0.043	0.8
30	0.521	0.029	0.9	0.464	0.031	1.2	0.681	0.015	0.5	0.539	0.04	0.9
40	0.515	0.029	0.9	0.433	0.038	1.3	0.682	0.017	0.5	0.556	0.038	0.8
50	0.513	0.032	0.9	0.42	0.04	1.4	0.678	0.017	0.5	0.542	0.038	0.8
60	0.51	0.035	1.0	0.42	0.047	1.4	0.672	0.019	0.5	0.535	0.037	0.9

CONDUCTIVITY, κ (mS cm^{-1})

-	DEAA			DEAH			MEAA			MEAH		
T, °C	X_{IL}	error	H_2O/PIL	X_{IL}	error	H_2O/PIL	X_{IL}	error	H_2O/PIL	X_{IL}	error	H_2O/PIL
25	0.243	0.01	3.1	0.366	0.043	1.7	0.29	0.019	2.4	0.149	0.005	5.7
30	0.241	0.012	3.1	0.371	0.044	1.7	0.288	0.017	2.5	0.151	0.006	5.6
40	0.245	0.015	3.1	0.389	0.057	1.6	0.291	0.018	2.4	0.156	0.007	5.4
50	0.335	0.022	2.0	0.522	0.056	0.9	0.298	0.017	2.4	0.157	0.007	5.4
60	0.34	0.02	1.9	0.539	0.047	0.9	0.303	0.017	2.3	0.158	0.007	5.3
65	0.34	0.02	1.9	0.551	0.046	0.8	0.307	0.017	2.3	0.14	0.016	6.1

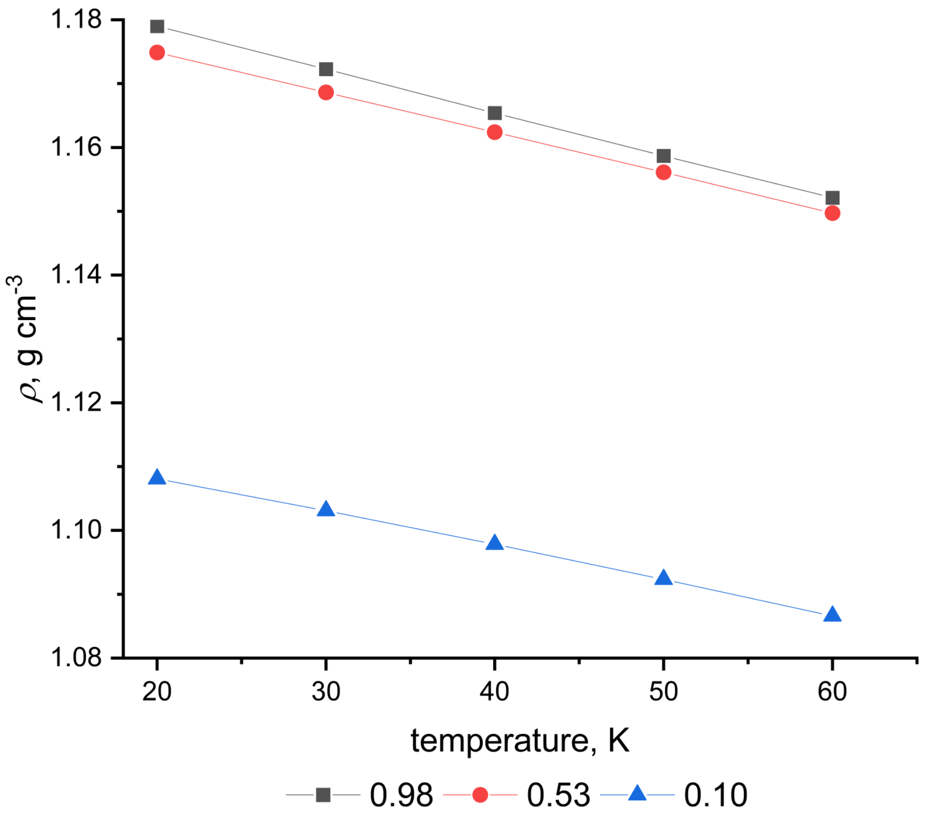

Figure S1: The density (ρ) of DEAA-H_2O mixtures with a varying ionic mole fraction (X_{IL}) presented as a function of the temperature)T, °C): $X_{\mathrm{DEAA}} = 0.98$ (black square); $X_{\mathrm{DEAA}} = 0.53$ (red dot); $X_{\mathrm{DEAA}} = 0.10$ (blue triangle).

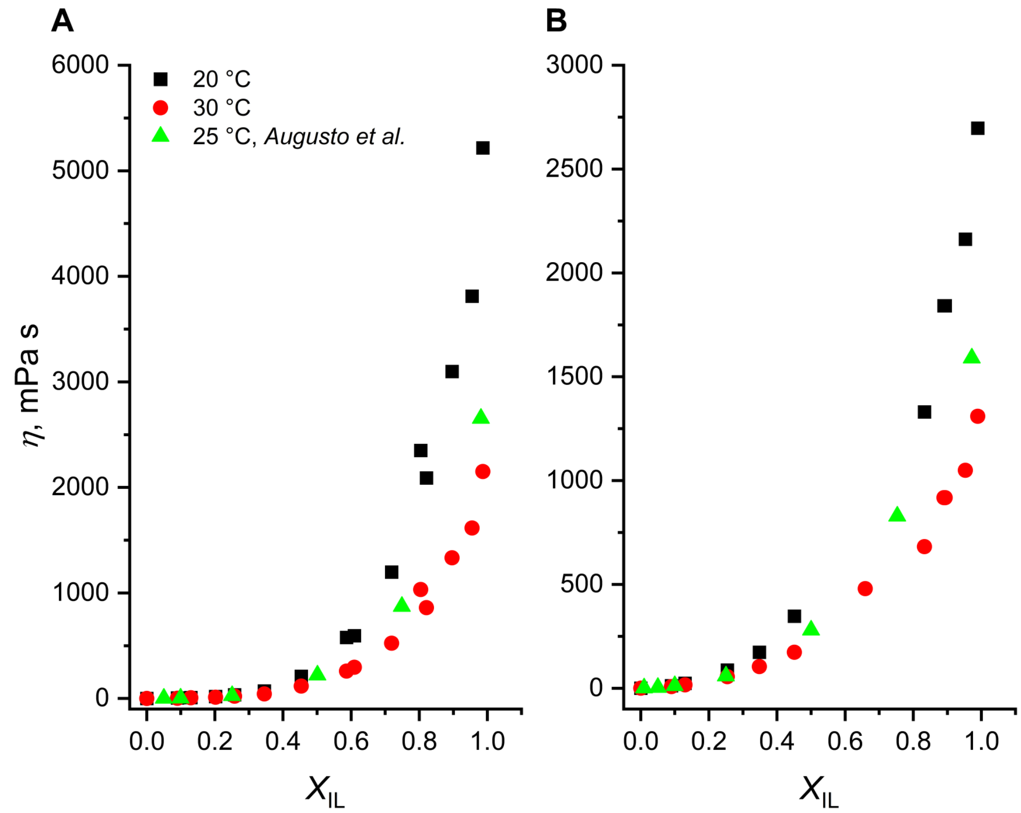

Figure S2: The viscosity (η) of MEAA-H_2O (**A**) and MEAH-H_2O (**B**) mixtures presented as a function of ionic liquid mole fraction (X_{IL}). For comparison, the experimental results at 20 °C (black square) and 30 °C (red dot) are paired with literature data for both, MEAA-H_2O and MEAH-H_2O adapted from *Augusto, et. al* (green triangle) [1].

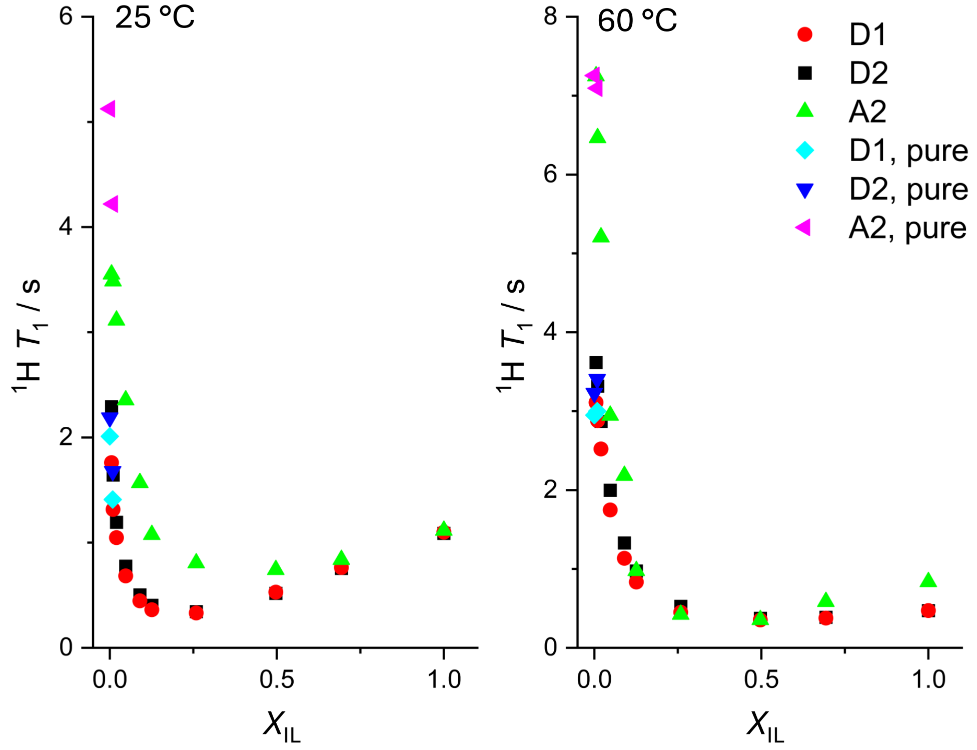

Figure S3: ^{1}H T_1 (s) determined in diethanolammonium acetate (DEAA) – water mixtures and represented as a function of the mole fraction of the ionic liquid (X_{IL}). The ^{1}H T_1 were measured at 25 °C (left) and 60 °C (right). Additional data (light and dark blue, pink, labeled as pure) present the ^{1}H T_1 of protons in the cation or anion separately, in the single-compound solution. The labeling scheme is presented in Figure 1.

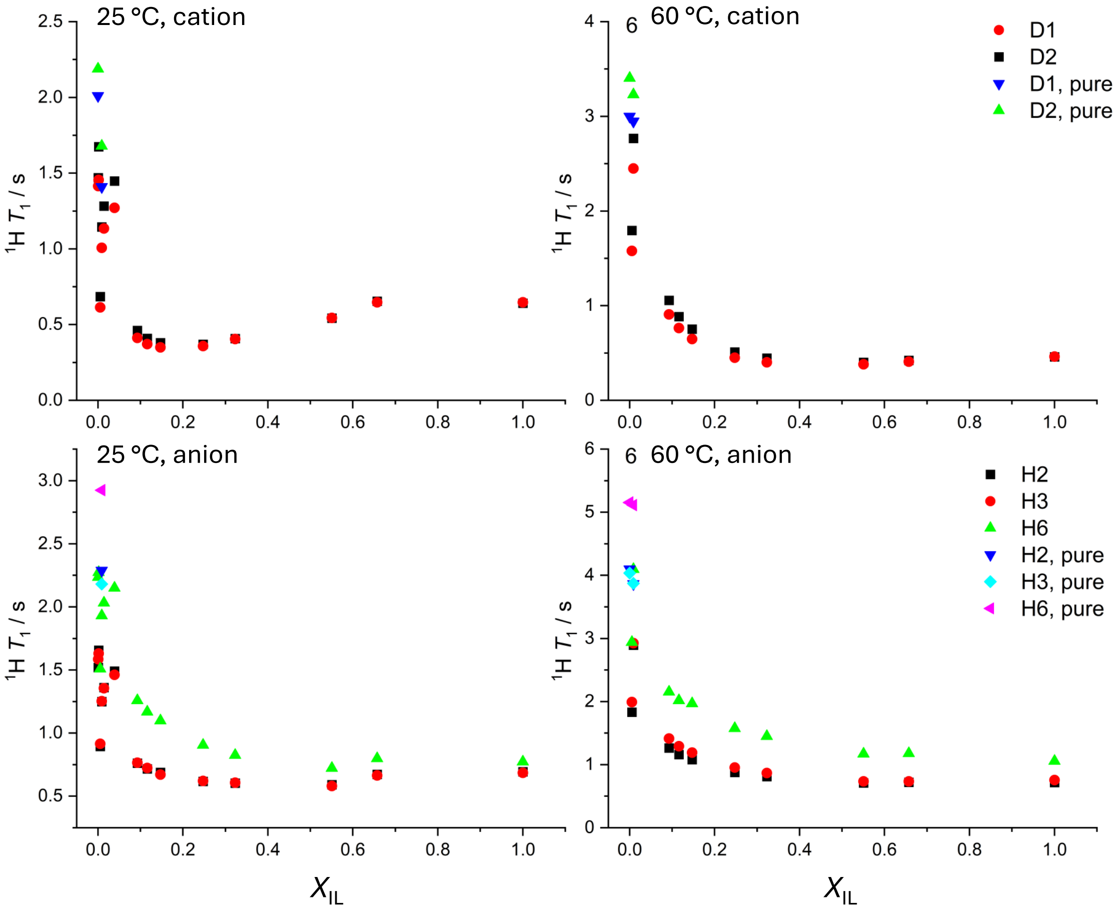

Figure S4: ^{1}H T_1 (s) determined in diethanolammonium hexanoate (DEAH) – water mixtures and represented as a function of the mole fraction of the ionic liquid (IL) for the cation (top) and anion (bottom). The ^{1}H T_1 were measured at 25 °C (left) and 60 °C (right). Additional data (blue and green for the cation, light and dark blue, pink, all labeled as pure) present the ^{1}H T_1 of protons in the cation or anion separately, in the single-compound solution. The labeling scheme is presented in Figure 1.

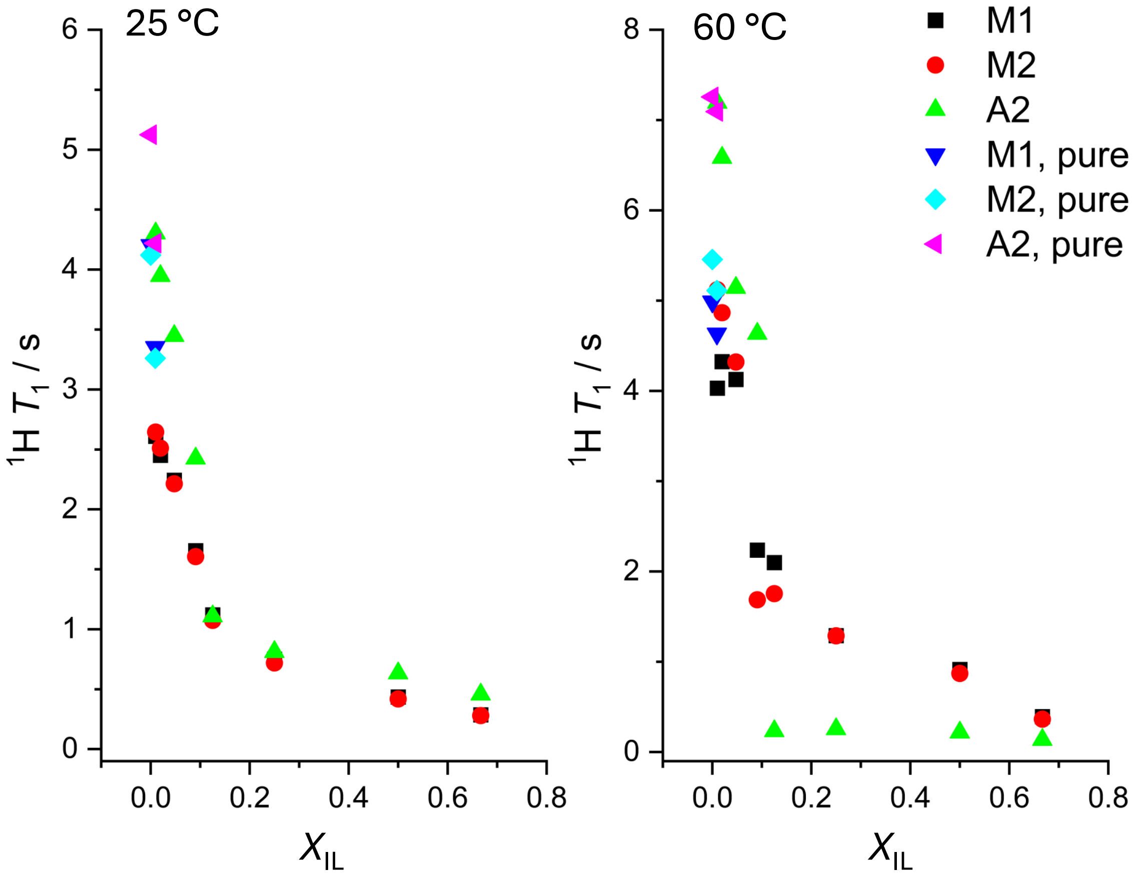

Figure S5: ^{1}H T_1 (s) determined in monoethanolammonium acetate (MEAA) – water mixtures and represented as a function of the mole fraction of the ionic liquid (IL). The ^{1}H T_1 were measured at 25 °C (left) and 60 °C (right). Additional data (light and dark blue, pink, labeled as pure) present the ^{1}H T_1 of protons in the cation or anion separately, in the single-compound solution. The labeling scheme is presented in Figure 1.

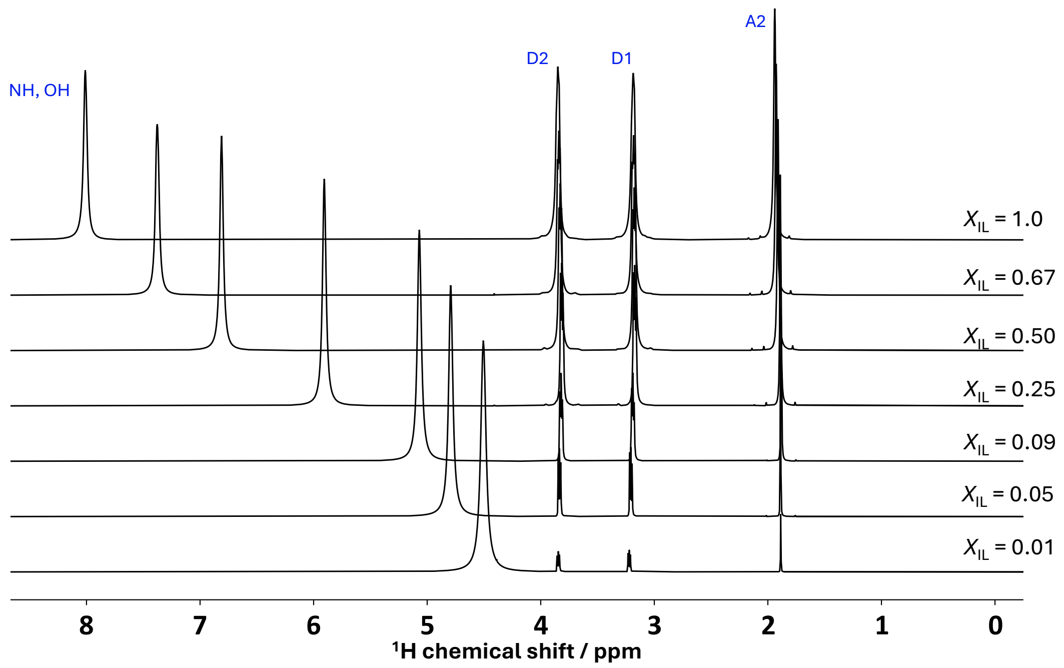

Figure S6: ^{1}H 1D NMR spectra acquired at 333 K in a series of DEAA-H_2O binary mixtures. The mole fraction of ionic liquid X_{IL} varies between 0.01 and 1.0, the latter representing pure DEAA. The labeling scheme is presented in Figure 1.

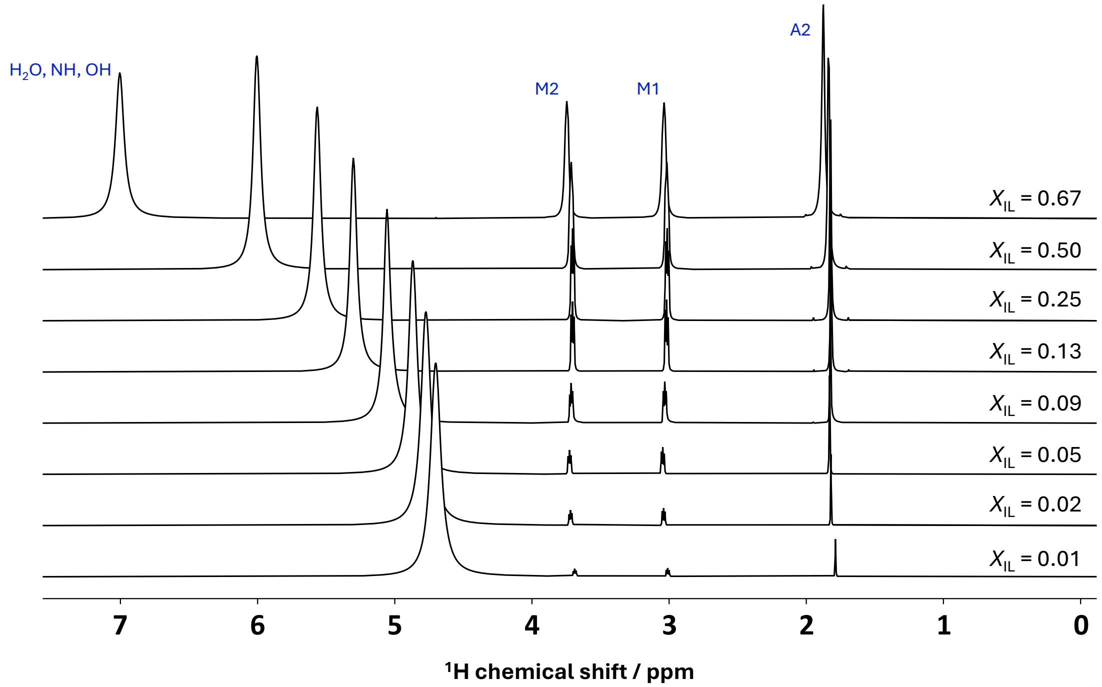

Figure S7: ^{1}H 1D NMR spectra acquired at 298 K in a series of MEAA-H_2O binary mixtures. The mole fraction of ionic liquid X_{IL} varies between 0.01 and 0.67. The labeling scheme is presented in Figure 1.

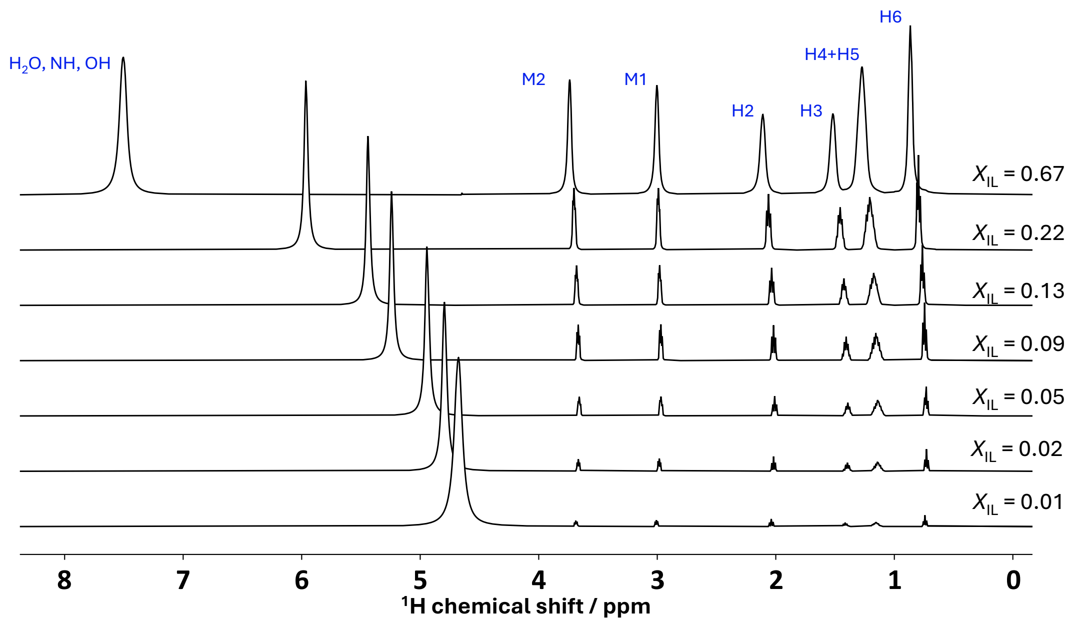

Figure S8: ^{1}H 1D NMR spectra acquired at 298 K in a series of MEAH-H_2O binary mixtures. The mole fraction of ionic liquid X_{IL} varies between 0.01 and 0.67. The labeling scheme is presented in Figure 1.

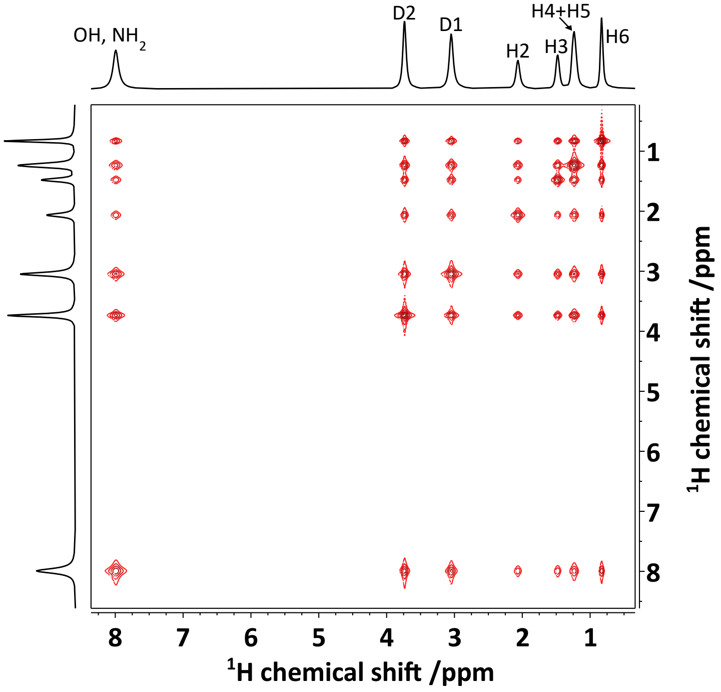

Figure S9: ^{1}H – ^{1}H 2D NOESY spectrum of pure diethanolammonium hexanoate (DEAH), measured at 298 K and mixing time 400 ms. The labeling scheme is presented in Figure 1.

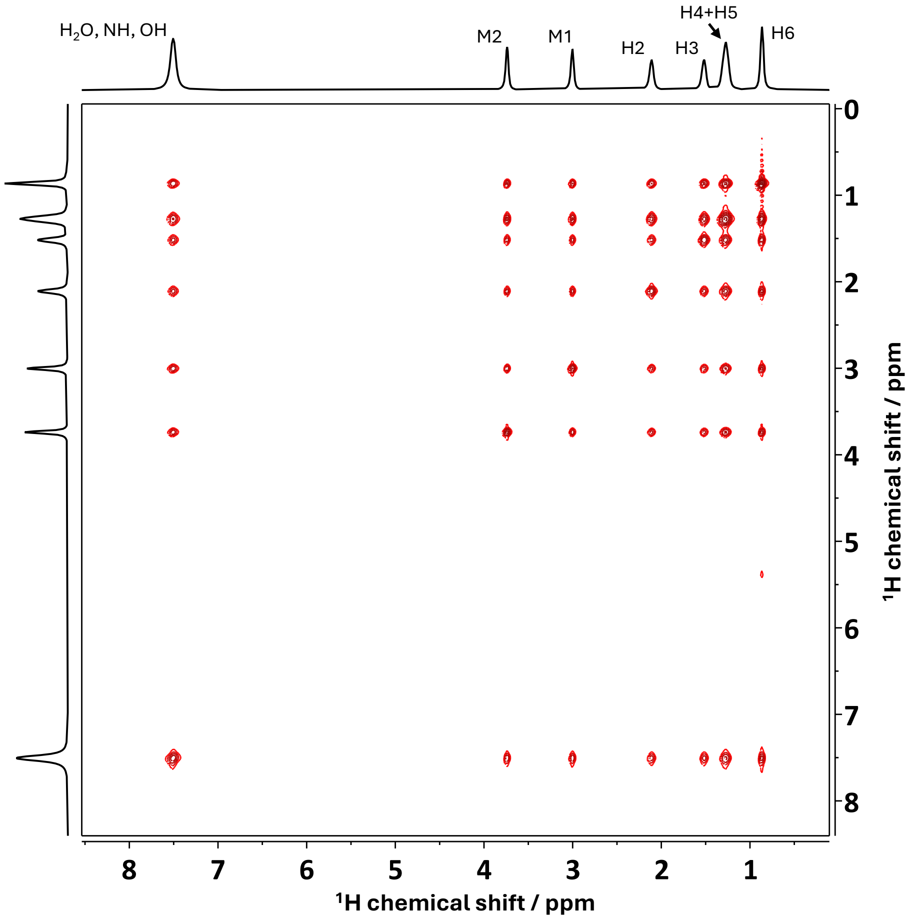

Figure S10: ^{1}H – ^{1}H 2D NOESY spectrum of pure ethanolammonium hexanoate (MEAH), measured at 298 K and mixing time 400 ms. The labeling scheme is presented in Figure 1.

A

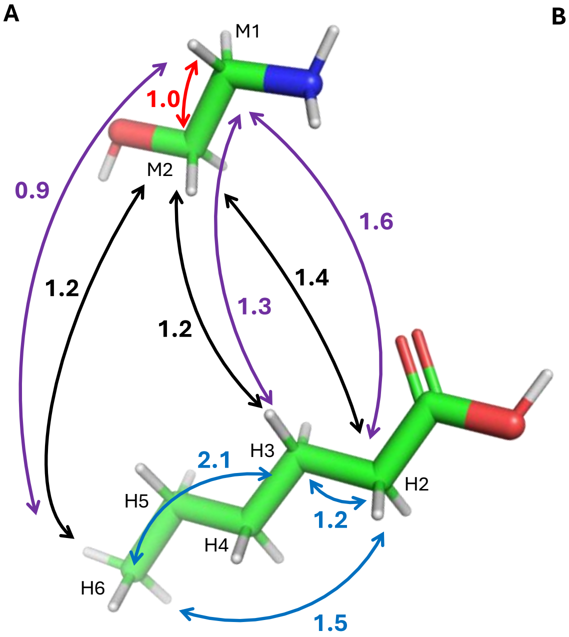

B

	X_{IL} = 0.67	X_{IL} = 0.44
**M1-M2**	1.0	1.0
**M2-H2**	0.9	2.6
**M2-H3**	0.8	2.5
**M2-H6**	0.9	2.3
**M1-H2**	0.8	2.7
**M1-H3**	1.0	x
**M1-H6**	1.1	2.4
**H2-H3**	1.1	4.7
**H2-H6**	1.5	2.8
**H3-H6**	1.3	4.7

Figure S11: Dipolar couplings, σ (s^{-1}), determined at 298 K in monoethanolammonium hexanoate (MEAH) – water binary mixtures with varying mole fraction of ionic liquid (IL) X_{IL}. **(A)** A visual representation of all dipolar couplings determined between the cation (MEA) and anion (H) molecules, and normalized for the intramolecular M1-M2 interaction. **(B)** Tabularized values of all dipolar couplings presented in graphic (A) at two mole fractions of ionic liquid: $X_{\mathrm{IL}} = 0.67$ and 0.44. Red: reference M1-M2 interaction; black: interactions of M1 in the cation; purple: interactions of M2 in the cation; blue: inter- and intramolecular interactions between protons in the anion.

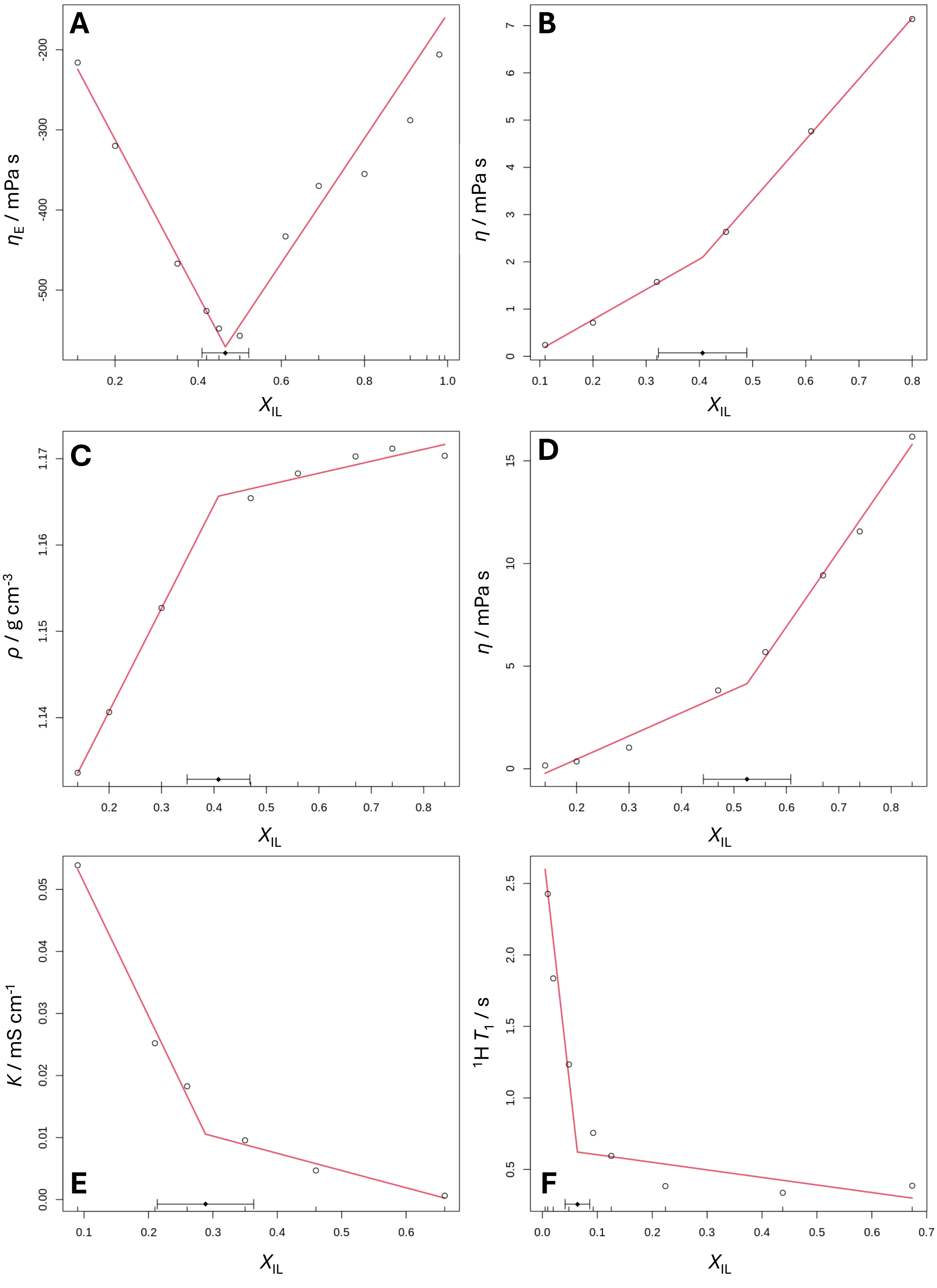

Figure S12: An example of the method of continuous variation applied to different variables measured in different protic ionic liquids and represented as a function of the mole fraction of the ionic liquid (IL) X_{IL}. **A:** excess viscosity (η_E, mPa s) determined in DEAH-H_2O mixtures; **B:** viscosity (η, mPaS) in DEAH-H_2O mixtures; **C:** density (ρ, g cm^{-3}) in DEAA-H_2O mixtures; **D:** η in DEAA-H_2O mixtures; **E:** conductivity (κ, mS cm^{-1}) in MEAA-H_2O mixtures, and **F:** ^{1}H T_1 relaxation time of proton labeled M2 in MEAH-H_2O mixtures. The red line represents the linear fit to the data points (black circles). The estimated break point value and corresponding uncertainty are presented at the bottom (black dot and bars).

Supplemental Appendix S1: R script used to carry out the estimation of critical aggregation concentrations.

```
## Example script used to estimate critical aggregation concentration (CAC)

## 1. Requirements:
# R (version 3.5 or above)

## 1. install the 'segmented' package
# install.packages("segmented") # uncomment this line
# to install the 'segmented' package if you do not have it already
library(segmented)

## 2. Load data from the file "example_data.csv"
# example_data.csv contains the columns 'x_var' and 'y_var'
# (e.g.: Mole fraction of IL and excess viscosity, respectively)
df = read.table("example_data.csv", sep=",", header=TRUE)

## 3. Fit linear regression model of 'y_var' with a single break point in 'x_var'.
fit.lm <- lm(y_var~x_var,data=df)
fit.seg <- segmented(fit.lm, seg.Z=~x_var, npsi = 1)
fit.seg$psi # print estimated break point

## 4. Plot model fit with estimated break point
plot(fit.seg, ylab="y_var") # plot fit
lines(fit.seg) # plot estimated break point
points(df$x_var,df$y_var) # overlay the measured data points

# As an example, when 'y_var' = excess viscosity
# determined in diethanolammonium hexanoate (DEAH) - water mixtures
# and 'x_var' = the mole fraction of the ionic liquid,
# the analysis reproduces the corresponding results in Table S6, and Figure S8.
```

1

References

[1] Ferrari Felipe Augusto et al. "Physicochemical Characterization of Two Protic Hydroxyethylammonium Carboxylate Ionic Liquids in Water and Their Mixture". In: *Journal of Chemical & Engineering Data* 67.6 (2022), pp. 1309–1325. DOI: 10.1021/acs.jced.1c00687.
